# A Novel Function of Na_V_ Channel β3 Subunit in Endothelial Cell Alignment Through Autophagy Modulation

**DOI:** 10.1096/fj.202401558RR

**Published:** 2025-05-30

**Authors:** Léa Réthoré, Anne‐Laure Guihot, Linda Grimaud, Coralyne Proux, Benjamin Barré, François Guillonneau, Catherine Guette, Alice Boissard, Cécile Henry, Jérôme Cayon, Rodolphe Perrot, Daniel Henrion, Christian Legros, Claire Legendre

**Affiliations:** ^1^ INSERM, CNRS, MITOVASC, Equipe CarME, SFR ICAT, Univ Angers Angers France; ^2^ Univ Angers Angers France; ^3^ Institut de Cancérologie de l'Ouest (ICO) Angers France; ^4^ Prot'ICO (ICO Proteomic Core Facility) Angers France; ^5^ INSERM, CNRS, CRCI2NA, Nantes Université, Univ Angers Angers France; ^6^ SFR ICAT, PACeM (Plateforme d'Analyse Cellulaire et Moléculaire), Univ Angers Angers France; ^7^ SFR ICAT, SCIAM (Service Commun d'Imageries et d'Analyses Microscopiques), Univ Angers Angers France

**Keywords:** autophagy, endothelial cell, mechanosignaling, Na_V_β3, *SCN3B*, shear stress

## Abstract

Endothelial cells (EC) play a pivotal role in vascular homeostasis. By sensing shear stress generated by blood flow, EC endorse vasculoprotection through mechanotransduction signaling pathways. Various ion channels are involved in mechanosignaling, and here, we investigated the endothelial voltage‐gated Na^+^ channels (Na_V_ channels), since their mechanosensitivity has been previously demonstrated in cardiomyocytes. First, we showed that EC from aorta (TeloHAEC) behave as EC from umbilical vein (HUVEC) under laminar shear stress (LSS). For both EC models, cell alignment and elongation occurred with the activation of the KLF2/KLF4 atheroprotective signaling pathways. We found that LSS decreased the expression of *SCN5A*, encoding Na_V_1.5, while LSS increased that of *SCN3B*, encoding Na_V_β3. We demonstrated that the KLF4 transcription factor is involved in *SCN3B* expression under both static and LSS conditions. Interestingly, *SCN3B* silencing impaired EC alignment induced by LSS. The characterization of Na_V_β3 interactome by coimmunoprecipitation and proteomic analysis revealed that mTOR, implicated in autophagy, binds to Na_V_β3. This result was evidenced by the colocalization between Na_V_β3 and mTOR inside cells. Moreover, we showed that *SCN3B* silencing led to the decrease in LC3B expression and the number of LC3B positive autophagosomes. Furthermore, we showed that Na_V_β3 is retained within the cell and colocalized with LAMP1 and LC3B. Finally, we found that resveratrol, a stimulating‐autophagy and vasculoprotective molecule, induced KLF4 together with Na_V_β3 expression. Altogether, our findings highlight a novel role of Na_V_β3 in endothelial function and cell alignment as an actor in shear stress vasculoprotective intracellular pathway through autophagy modulation.

## Graphical abstract

NaVβ3 expression is increased by laminar shear stress (LSS) through KLF4 induction (1). Na_V_β3 is retained within the cell and interacts with mTOR protein and colocalizes partially with LAMP1 and LC3B, lysosome and autophagosome markers, respectively (2). Since SiRNA SCN3B induces the decrease of LC3B expression and cell alignment in response to LSS (3), we assume that Na_V_β3 is involved in autophagic flux (4) which contributes to cell alignment and vasculoprotection.

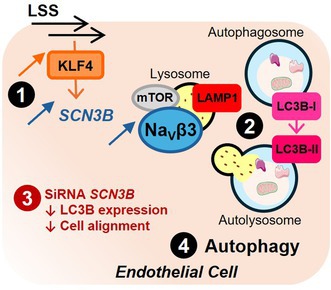

**Abbreviations**
ANOVA: analysis of variance
B2M: Beta‐2‐Microglobulin
BSA: bovine serum albumin
Cq: cycle quantitative
DAPI: 4′,6‐diamidino‐2‐phenylindole
DDA: data‐dependent acquisition
ddPCR: droplet digital polymerase chain reaction
DMEM: Dulbecco's modified Eagle medium
DRG: Dorsal Root Ganglion neurons
DTT: dithiothreitol
EBM‐2: endothelial basal medium 2
EBM‐MV2: endothelial basal medium from microvascular vessels
EC: endothelial cells
EDTA: ethylenediaminetetraacetic acid
EGM‐2: endothelial growth medium 2
EGM‐MV2: endothelial growth medium from microvascular vessels
eNOS: endothelial nitric oxide synthase
FDR: false discovery rate
FKBP12: FK506‐binding protein 12
FT: flow through
GAPDH: glyceraldehyde‐3‐phosphate dehydrogenase
HAEC: human aortic endothelial cell
HSC70: heat shock cognate protein 70
HUVEC: human vein endothelial cell
Ig‐CAM: immunoglobulin cellular adhesion molecule
IP: immunoprecipitation
KLF: krüppel‐like transcription factor
LC3B or MAP1LC3B: microtubule‐associated protein 1 light chain 3 beta M
LC–MS/MS: liquid chromatography coupled to tandem mass spectrometry
LSS: laminar shear stress
MMTS: S‐Methyl methanethiosulfonate
mTOR: mammalian target of rapamycin
mTORC: mTOR complex
Na_V_ channels: voltage‐gated Na^+^ Channels
PBS: phosphate‐buffered saline
PCC: Pearson's correlation coefficient
PECAM1: platelet endothelial cell adhesion molecule
PEI: polyethylenimine
PFM: position frequency matrix
RSV: Resveratrol
TBP: tata‐box protein
TRPV4: transient receptor potential vanilloid 4
TTX: tetrodotoxin
UBC: ubiquitin C

## 1 Introduction

The endothelium is defined as a single layer of endothelial cells (EC) separating blood from the vessel wall. It plays a protective role in vascular homeostasis [1]. Indeed, the EC function allows the regulation of vasomotor tone in response to blood flow, as well as the preservation of blood vessel wall integrity and the regulation of angiogenesis [1]. Thus, endothelial function, via the vasculoprotective effects, preserves cardiovascular health, and endothelial dysfunction is a predictive factor for the occurrence of cardiovascular events including arterial hypertension and atherosclerosis. The endothelium is therefore a priority preventive and therapeutic target organ to reduce cardiovascular risk [2].

The key stimulus for EC function and vasculoprotection is the shear stress generated by laminar blood flow, which appears when the vessels are linear [3]. This laminar shear stress (LSS) ranges from 10 to 60 dyn/cm^2^ in the arterial vascular network [4] and actively protects against vascular diseases through the activation of the atheroprotective molecular signaling pathways [5]. Among the flow‐dependent transcription factors, Krüppel‐like transcription factor (KLF) including KLF2 [6, 7] and KLF4 [8] are master regulators in the response of EC to LSS. KLF activation governs EC alignment in the direction of flow [9], associated with the structural reorganization of the actin cytoskeleton [9, 10]. KLF signaling pathway triggers also the expression of genes necessary for EC stability and quiescence, such as the endothelial nitric oxide synthase eNOS encoded by the *NOS3* gene, which promotes vasodilation and prevents inflammation [11].

Another mechanism involved in LSS‐mediated endothelial vasculoprotection is autophagy. Autophagy refers to a set of pathways by which cytoplasmic content is sequestered in double‐membrane vesicles called autophagosomes [12]. These autophagosomes are then subsequently degraded by fusion with lysosomes and generate autolysosomes, leading to the digestion and liberation of macromolecules and metabolites that can be reused [12]. The autophagy process allows for cellular homeostasis maintenance [12] and participates in the vasculoprotection under LSS [13, 14, 15]. Autophagy also participates in the protective effects of vasculoprotective molecules such as resveratrol [16, 17]. Among the autophagic pathways described in EC, the serine/threonine kinase mTOR (mammalian target of rapamycin) is activated by low pathological shear stress, leading to inhibition of autophagy and subsequently to endothelial dysfunction [15]. The biogenesis of autophagosomes is governed by the last effector of autophagy, the microtubule‐associated protein 1 light chain 3 beta MAP1LC3B, also called LC3B. Under LSS, the autophagy process is essential for cell alignment induced by LSS, and it prevents EC apoptosis, senescence, and inflammation [13, 14, 15] and regulates flow‐mediated eNOS activation [16], linking cell recycling processes to the transduction of hemodynamic forces.

Indeed, LSS are sensed by specific membrane proteins that transduce mechanical forces into intracellular signals. These proteins are called mechanosensors or mechanotransducers, including mechanosensitive ion channels, such as the K^+^ channels Kir2.1, the nonselective cation channels transient receptor potential vanilloid 4, TRPV4, and Piezo1 [17]. The voltage‐gated Na^+^ channels (Na_V_ channels) that are expressed in human vein endothelial cell HUVEC [20] modulate ERK signaling upon LSS in vitro [21]. Our recent data have demonstrated that Na_V_ channels are involved in shear stress mechanosignaling, since the selective potent inhibitor of Na_V_ channels, tetrodotoxin (TTX), potentiates flow‐mediated dilation response in resistance arteries [22].

Na_V_ channels are composed of two subunits, an α‐subunit that forms a Na^+^ selective pore and an auxiliary β‐subunit, belonging to the immunoglobulin cellular adhesion molecule Ig‐CAM family, as chaperone and gating regulator [21]. In the mammal genome, nine genes (*SCN1‐5A* and *SCN8‐11A*) encode as many as Na_V_ channel α‐subunit isoforms (Na_V_1.1 to Na_V_1.9) and four genes (*SCN1B* to *SCN4B*) encode Na_V_ channel β‐subunit isoforms (Na_V_β1 to Na_V_β4, and also Na_V_β1B isoform resulting from alternative splicing of *SCN1B*) [22]. These ion channels are key components of membrane excitability and are mainly expressed in neurons, myocytes, and cardiomyocytes, allowing action potential generation and propagation [23]. However, for the past 2 decades, noncanonical roles of both α‐ and β‐subunit isoforms of Na_V_ channels have emerged [24], such as phagocytosis [25] and migration and proliferation in cancer cells [26]. Mechanosensitivity of Na_V_ channels has been clearly demonstrated in cardiomyocytes and intestinal cells [27, 28, 29, 30, 31], through an electrical‐contraction coupling mechanism, mediated by both Na_V_β1 and Na_V_β3 subunits [28].

Here, we aimed to better understand the role of Na_V_ channels in LSS. By investigating the expression of Na_V_ channels in EC submitted in vitro to LSS, we focused our attention on Na_V_β3, encoded by the *SCN3B* gene, in two human EC models, one obtained from umbilical vein (HUVEC), and the other from the aorta (TeloHAEC). We examined the correlation between *SCN3B* expression and cell alignment. Notably, we studied the relationship between *SCN3B* expression and KLF4, a vasculoprotective transcription factor, and the effects of *SCN3B* silencing on mTOR and LC3B expression, which govern autophagosome formation. Furthermore, we investigated in which intracellular compartments Na_V_β3 is expressed, in comparison with LAMP1, LC3B, and mTOR, to further address its role in autophagy. In addition, we explored the effect of resveratrol, a vasculoprotective molecule, on Na_V_β3 expression.

## 2 Materials and Methods

### 2.1 Chemical Reagents

All reagents and solvents were obtained from Sigma‐Aldrich Merck (Saint‐Louis, MO, USA) or Thermo Fisher Scientific (Waltham, MA, USA) except RapiGest SF Surfactant, which was obtained from Waters Corporation (186001861, Milford, MA, USA), and resveratrol (RSV) from Abcam (Cambridge, UK).

### 2.2 Cell Lines and Treatments

Human umbilical vein endothelial cells (HUVEC) obtained from PromoCell (C‐12203; Heidelberg Germany) were cultured from passage 2 until passage 5 at 37°C with 5% CO_2_ in endothelial basal medium 2 (EBM‐2; C‐22211; PromoCell) supplemented with the SupplementMix (C‐39216; PromoCell) called endothelial growth medium 2 (EGM‐2). Human aortic endothelial cells (TeloHAEC) (CRL‐4052; LGC Standards, Molsheim, France) were cultured from passage 2 until passage 25 at 37°C with 5% CO_2_ in endothelial basal medium from microvascular vessels (EBM‐MV2; C‐22221; PromoCell) supplemented with the SupplementMix (C‐39226; PromoCell) called endothelial growth medium 2 from microvascular vessels (EGM‐MV2). HUVEC and TeloHAEC were routinely cultivated in Flask T175 in EGM‐2 or EGM‐MV2 medium, respectively, and when 80% confluence was reached, split in a ratio of 1/5 (HUVEC) or 1/10 (TeloHAEC) in a new Flask T175 using the DetachKit (C‐41210; PromoCell). COS‐7 cells were cultivated in a Dulbecco's modified Eagle medium (DMEM)/F12 medium (P04‐41550, Lonza, Basel, Switzerland) supplemented with 10% fetal bovine serum (Lonza), 1 mM L‐glutamine, 1 mM of penicillin/streptomycin, and 1 mM of pyruvate in an incubator with 5% CO_2_ at 37°C. COS‐7 cells were routinely cultivated in Flask T75, and when 80% confluence was reached, split in a ratio of 1/10 in a new Flask T75. For resveratrol (RSV) treatment, HUVEC or TeloHAEC were plated at 80% confluence in 6‐well plates and incubated the next day with 100 μM of RSV for 48 h in EGM‐2 or EGM‐MV2 using RSV solution stock of 100 mM freshly dissolved in DMSO. Control cells (CTL) were treated with 0.1% of DMSO.

### 2.3 In Vitro Shear Stress Procedure

EC were seeded at 90% confluence (350 000 cells) onto μ‐slides 0.4 or 0.8 Luer (80176; Ibidi GmbH, Munich, Germany) 24 h before shear stress experiments. The μ‐slides were then connected to the Ibidi pump system (10902) with a perfusion set of 15 cm, inner diameter 1.6 mm (10962) according to the Ibidi manufacturer's instructions (AN 13: HUVECs under Perfusion). The slides were incubated at 37°C with 5% CO_2_. Cells were exposed to a laminar unilateral shear stress (LSS) of 20 dyn/cm^2^, with these flow parameters: 31.9 mbar pressure, 21.7 mL/min flow rate, 0.007 dyn × s/cm^2^ viscosity for 24 h or 4 days. Static condition corresponded to cells cultivated onto Ibidi μ‐slides 0.4 Luer without shear stress application. For this static condition, cell medium was changed every day.

### 2.4 Cell Alignment and Elongation Quantifications

Cell alignment was assessed using the local gradient orientation method with the directionality plugins in ImageJ—Fiji software. Cell alignment is determined using the angle formed between the orientation vector of the flow direction (corresponding to an angle value of 0°) and the main axis of cells, called cell orientation in degree. The distribution of cells within each angle value (cell orientation in degree) is given as the cell amount value in arbitrary units. Histograms represent the quantity of cell alignment (in arbitrary unit) between the angle values of −5° to +5° to quantify all cells aligned to the flow direction.

Quantification of cell length and width giving the elongation factor (cell length along flow direction divided by cell width) was performed with ImageJ software, where for each technical replicate, 15 cells per field were counted for each culture condition (static or LSS).

### 2.5 SiRNA or Plasmid Transfection

TeloHAEC were transfected with a custom *KLF4* SiRNA Oligo Duplex sequence called SiRNA *KLF4* or SiKLF4 (AGAUUAAGCAAGAGGCGGU)(UU)—Eurofins Genomics, Ebersberg, Germany—or with *SCN3B* siRNA Oligo Duplex sequence (Locus ID 55800—SR310954) called SiRNA *SCN3B* or SiSCN3B (CCUAGUCACUGCAAAUGUAUCUGAA—OriGene, Rockville, MD, USA) or with a universal scrambled negative control SiRNA duplex (AGGUAGUGUAAUCGCCUUG(UU)—OriGene, Rockville, MD, USA) called SiRNA CTL or SiCTL, using Lipofectamine RNAiMAX Reagent (Cat No. 13778‐150, Invitrogen Life technologies, Thermo Fisher Scientific). Briefly, TeloHAEC were seeded at a density of 500 000 cells/well in a six‐well plate. The day after, cells were transfected with a mix of 1:1 *v*/*v* lipofectamine: SiRNA (10 nM final) for 24 h. Cells were then trypsinized and seeded at 90% confluence (350 000 cells) onto μ‐slides 0.4 Luer 24 h before the shear stress experiment for 24 h.

The expression plasmid, pMyc‐SCN3B containing the ORF of *SCN3B* with a sequence encoding Myc tag (10 amino acid EQKLISEEDL) was used in this study (pCMV3‐SP‐N‐Myc‐SCN3B, Cat No. HG13897‐NM, Sino Biological Europe, Düsseldorf, Germany). The vector pCTL served as the negative control vector (pCMV3‐SP‐N‐Myc from Sino Biological Europe). TeloHAEC were transfected using Lipofectamine 2000 Reagent (Invitrogen Life technologies), whereas COS‐7 cells were reverse transfected using Polyethylenimine (PEI, Cat No. 23966, Polysciences) according to the manufacturer's instructions. Cells were transfected with 1 μg of plasmid per 100 000 cells (TeloHAEC) or 300 000 cells (COS‐7). TeloHAEC were also transfected with pPB[Exp]‐EGFP/Puro‐CMV>hKLF4 (NM_001314052.2), pKLF4 plasmid, (VectorBuilder, Neu‐Isenburg, Germany) using Lipofectamine LTX with Plus Reagent (Invitrogen Life technologies) with 1 μg of plasmid per 100 000 cells for 24 h. The vector (pPB[Exp]‐EGFP/Puro‐CAG>ORF Stuffer, Cat No. VB010000‐9294rpr, VectorBuilder) was used as the negative control.

### 2.6 Reverse Transcription (RT) and Real‐Time Quantitative Polymerase Chain Reaction (RT‐qPCR)

After treatments, cells were washed with ice‐cold PBS, and total RNA was extracted using the RNeasy micro kit (Qiagen, Courtaboeuf, France) according to the manufacturer's instructions. In total, 500 ng to 1 μg of total RNA was processed for cDNA synthesis using random hexamers and the QuantiTect Reverse Transcription kit (Qiagen). PCR assays were assessed on a LightCycler 480 Instrument II (Roche, Meylan, France) using Sybr Select Master Mix (Applied Biosystems, Thermo Fisher Scientific) or Power Sybr Select Master Mix and 10 ng of cDNA in duplicate, and gene‐specific primers (Table S1) previously designed using the PrimerQuest Tool on Integrated DNA Technologies (IDT) and validated by testing PCR efficiency using a standard curve as per MIQE guidelines [32]. Amplification specificity was confirmed by one peak‐melting curve at the end of the amplification process. Relative quantification of gene expression was normalized to the mean of the expression of two validated housekeeping genes TBP (Tata‐box protein) and/or UBC (Ubiquitin C) and/or B2M (Beta‐2‐Microglobulin) and by using the 2^−ΔCq^ or 2^−ΔΔCq^ method, where Cq is the Cycle quantitative [33].

### 2.7 Droplet Digital Polymerase Chain Reaction (ddPCR)

The ddPCR experiments were performed using the QX200 ddPCR System (BioRad, Hercules, CA, USA), using 20 ng of cDNA normalized in quantity during the RT step. No‐template controls and positive controls (i.e., HEK293 transfected with pMyc‐SCN3B) were also tested. All samples were tested in duplicate. Each reaction was set up per the manufacturer's instructions in a 22 μL sample volume containing 11 μL of 2 × ddPCR Evagreen supermix (Bio‐Rad Laboratories Inc), 150 nM *SCN3B* specific forward primer AAGAGAGAGGAGGTGGAGGC and reverse primer GTGGCCATTCCGATACTCGT. Droplets were generated in a QX200 manual droplet generator according to the manufacturer's instructions, giving a final volume of 40 μL. DNA amplification was carried out using the following PCR program: initial denaturation at 95°C for 5 min, amplification with 40 cycles at 95°C for 30 s and 60°C for 1 min followed by a final signal stabilization step at 4°C for 5 min and 90°C for 5 min (C1000 Touch thermal cycler; Bio‐Rad Laboratories Inc). Finally, droplets were analyzed in a QX200 droplet reader with Quantasoft software (Bio‐Rad Laboratories Inc), and data analysis of ddPCR files, including Poisson distribution analysis, was performed with QuantaSoft Software Standard Edition (version 1.2, Bio‐Rad Laboratories Inc). The threshold line differentiating positive from negative calls was determined automatically by the software and manually adjusted when necessary, using the signals in no‐template controls and positive control, as a guide. The minimum number of accepted droplets for quantification was 8500. Quantitative ddPCR data were used to calculate *SCN3B* concentrations in copy/μl of sample, taking into consideration dilution factors.

### 2.8 Western Blotting

After treatments, cells were washed with ice‐cold PBS and lysed at 4°C in RIPA buffer (50 mM Tris–HCl, 150 mM NaCl, 12 mM sodium deoxycholate, 0.1% SDS, 1% Triton X‐100, pH 8) supplemented with Halt Protease and Phosphatase Inhibitor Cocktail and EDTA (78444, Thermo Fisher Scientific). Cell lysates were centrifuged for 20 min at 14 000 rpm at 4°C, and supernatants were collected. After protein quantification (Pierce BCA Protein Assay Kit, Cat No. 23235), 10 to 50 μg of proteins were separated by 8% to 12% SDS‐Polyacrylamide Gel Electrophoresis or 4%–20% precast polyacrylamide gel (Bio‐Rad, Marnes‐la‐Coquette, France) and were transferred onto a nitrocellulose membrane 0.22 μM (Thermo Fisher Scientific). The primary antibodies diluted in TBST 5% (w/v) BSA used were anti‐eNOS/NOS Type III (1/500, mouse, 610297, BD Biosciences), anti‐KLF2 (1/1000, rabbit, HPA055964, Atlas Antibodies), anti‐KLF4 (1/1000, rabbit, 4038, Cell Signaling Technology), anti‐Myc tag [9E10] (1/1000, mouse, ab32, Abcam, Cambridge, UK), anti‐Na_V_1.5 (1/500, rabbit, ASC‐005, Alomone Labs), anti‐Na_V_1.6 (1/500, rabbit, ASC‐009, Alomone labs), anti‐Na_V_1.7 (1/500, mouse, 75‐103, NeuroMab), anti‐Na_V_β1 (1/1000, rabbit, 13950, Cell Signaling), anti‐Na_V_β3 (1/1000, rabbit, PA5‐87803 or PA5‐41403, Thermo Fisher Scientific or Ab4855, Abcam), anti‐mTOR (1/1000, rabbit, 2972, Cell Signaling Technology), anti‐P‐mTOR 2448 (1/1000, rabbit, 2971, Cell Signaling Technology), anti‐P‐mTOR 2481 (1/1000, rabbit, 2974, Cell Signaling Technology), anti‐LC3B (1/1000, rabbit, 3868, Cell Signaling Technology), and as loading controls: anti‐GAPDH (1/2000, mouse, A5316, Sigma or 1/2000, rabbit, 2118, Cell Signaling Technology (14C10)), anti‐β‐actin (1/2000, mouse, A5316, Sigma), or anti‐HSC70 (1/10 000, mouse, sc‐7298, Santa Cruz Biotechnology) were used as loading controls. Peroxidase‐conjugated secondary antibodies (Goat anti‐Mouse IgG, Cat No. 72‐8062, and Goat anti‐Rabbit IgG, Cat No. 72‐8073, Tonbo Biosciences) and Clarity Western ECL Substrate (170‐5061, Bio‐Rad) or maximum sensitivity substrate SuperSignal West Femto (34095, Thermo Fisher Scientific) were used before visualization using a LAS‐3000 imager (Fujifilm, Tokyo, Japan). The image acquisition was performed with Image Lab (Bio‐Rad) and protein normalization and densitometry analysis were done by adjusting background and comparing the adjusted volume of each band of the interest protein to the adjusted volume of the band of the loading control (HSC70 or GAPDH). Data of densitometry analysis is available in Table S2.

### 2.9 Immunofluorescence Staining and Colocalization Analysis

For immunofluorescence staining, TeloHAEC were cultivated onto μ‐slides VI 0.4 Luer (80606; Ibidi GmbH, Munich, Germany) previously coated with a gelatin‐based coating solution 0.1% (P06‐20410, PAN‐Biotech GmbH, Aidenbach, Germany) before seeding at 40 000 cells per channel. Cells were then subjected to SiRNA or plasmid transfection and cultivated under static or LSS conditions for 24 h. At the end of the experiments, cells were washed in PBS 1% at RT and fixed for 10 min with cold methanol 100% for LC3B staining or PFA 4% for the colocalization experiment and permeabilized for 10 min with 0.1% saponin in PBS (v/v) with 1% BSA, followed by 30 min blocking with 5% BSA, 0.01% saponin, and 100 mM glycine before probing with various primary antibodies at 4°C overnight. Antibodies used were as follows: anti‐Na_V_β3 (1/300, mouse, Alexa Fluor 488, S396‐29 clone, NBP2‐59318, Novus Biologicals, Toronto, Canada); anti‐LC3B (1/100, rabbit, 2775, Cell Signaling Technology), anti‐LAMP1 (1/200, rabbit, MA5‐29385, Thermo Fisher Scientific) and anti‐mTOR (1/100 rabbit, 2972, Cell Signaling Technology) in PBS/BSA 1%. After three washes in PBS/BSA 1%/saponin 0.01%, secondary antibodies Donkey antirabbit IgG (H + L) Alexa Fluor 568 (1/1000, A10042, Invitrogen, Thermo fisher Scientific) were then incubated for 45 min in PBS/BSA 1%. DAPI (10 μg/mL, D9542, Sigma) was incubated for 10 min in PBS, and slides were mounted with Vectashield Mounting Medium (H‐1000‐10, Eurobio Scientific, Les Ulis, France). Staining of negative controls was performed under the same conditions with, respectively, Mouse IgG2b isotype control (133303), Alexa Fluor 488, (1/300, IC0041G, Novus Biologicals, Toronto, Canada), and donkey antirabbit IgG (H + L) Alexa Fluor 568.

For LC3B immunofluorescence staining, fluorescent staining was visualized using confocal microscopy with inverted microscope ECLIPSE 2000‐E (Nikon, Champigny sur Marne, France) using a 60× water immersion objective (Nikon Plan Apo 60 ON 1.2) and CoolSNAP_HQ_^2^ camera controlled by Metamorph software (Molecular Devices, San Jose, CA, USA). Z series size was 0.3 μm with the MS‐2000 XYZ‐LE Microscope Stage controlled. The excitation was performed at 408 nm for 500 ms or 561 nm for 2000 ms, and the emission filter used has band‐pass respectively 460 ± 50 nm and 560 ± 50 nm.

For colocalization analysis, fluorescent staining was visualized using a Leica TCS SP8 AOBS confocal laser scanning microscope (Leica Microsystems, Wetzlar, Germany) equipped with a HC PL APO CS2 63X/ON 1.40 oil objective and gateable hybrid detectors (GaAsP). Images were acquired in the format 1024 × 1024 pixels, bit depth of 8, a scan speed of 400 Hz, and a X2 zoom using the LAS X software. Excitation was performed with a 488‐nm Argon laser (40 mW), a 561‐nm diode laser (20 mW) or a 633‐nm He‐Ne laser (10 mW). The bandpass for the detection of the emitted light was set, respectively, between 408 and 450 nm, 494 and 559 nm, and 564 and 630 nm. Z‐series optical sections were collected with a step size of 0.3 μm using a Super Z Galvo Type H stage.

Quantification of LC3B puncta dot (count) and intensity fluorescence has been determined using ImageJ—Fiji. Six to ten fields by conditions (SiCTL or SiSCN3B) and by experiment have been analyzed, and three independent experiments have been performed. To quantify the colocalization between Na_V_β3 and LC3B (marker of autophagosomes) or LAMP1 (marker of for late endosomes and lysosomes) or mTOR, the Jacob plugin available in ImageJ—Fiji was used to calculate the Pearson correlation coefficient (PCC) to quantify the degree of colocalization between fluorophores (Alexa Fluor 488 for Na_V_β3 and Alexa Fluor 568 for LC3B or LAMP1 or mTOR). Five to fifteen cells by conditions (Static or LSS) and by experiment have been analyzed, and three independent experiments have been performed.

### 2.10 Immunoprecipitation and Pull‐Down Assay

After transfection with pMyc‐SCN3B or pCTL vector for 24 h, TeloHAEC were washed with ice‐cold PBS, collected, and lysed with IP lysis buffer (20 mM Tris pH 7.4, 150 mM NaCl, 1% Triton) containing Halt Protease and Phosphatase Inhibitor Cocktail and EDTA (78444, Thermo Fisher Scientific). Cell lysates were centrifuged for 20 min at 14 000 rpm at 4°C, and supernatants, corresponding to input proteins, were collected for protein quantification (Pierce BCA Protein Assay Kit). For the immunocapture of Myc‐Navβ3, 500 μg of protein extract were incubated with washed binding control magnetic agarose beads (Cat No. bmab‐20, Chromotek) and rotated end‐over‐end for 1 h at 4°C. Then, this bead preparation was separated with a magnet until the supernatant was clear. This supernatant was incubated with washed Myc‐Trap magnetic agarose beads (Cat No. ytma‐10, Chromotek) overnight at 4°C. The next day, the Myc‐Trap agarose beads were captured until the supernatant was clear. The supernatant was collected and named Flow Through (FT). Beads were washed twice with IP lysis buffer. The first wash, named Wash, was collected for checking the absence of Navβ3. After washing with IP lysis buffer without Triton, beads were boiled in NH_4_CO_3_ buffer (50 mM pH 8) or with Laemmli buffer. The obtained supernatant (or elution named IP) was subjected to a western blot or mass spectrometry experiments. For the pulldown assay, COS‐7 cells were transfected with pMyc‐SCN3B plasmid or vector alone pCTL for 48 h. After collecting FT and Wash, 1 mg of freshly HUVEC whole protein lysate was incubated with magnetic beads for 1 h at 4°C in a rotating platform. Then, beads were washed twice with IP lysis buffer and once with IP lysis buffer without Triton. All protein samples (input, FT, wash, and IP) were analyzed by western blot.

### 2.11 Sample Preparation and Proteomic Analysis

For MS/MS analysis, immunoprecipitates were resuspended in 200 μL of Rapigest SF (Waters), and dithiothreitol (DTT) was added to a final concentration of 5 mM (AppliChem, Darmstadt, Germany). Samples were incubated in a thermo shaker at 95°C for 1 h, and sonication was performed twice using an ultrasonic processor (130 W, 20 KHz) (Thermo Fisher Scientific). Subsequently, cysteine residues were alkylated by adding 200 mM S‐Methyl methanethiosulfonate (MMTS) to a final concentration of 10 mM (incubated at 37°C for 10 min). Sequencing‐grade trypsin was added in a ratio ≥ 2 μg per 100 μg of protein (incubated at 37°C overnight). The reaction was stopped with formic acid (9% final concentration) and incubated at 37°C for 1 h, and the acid‐treated samples were centrifuged at 16 000 *g* for 10 min. Salts were removed from the supernatant and collected in new reaction microtubes using self‐packed C18 STAGE tips. Peptide concentrations were finally determined with the Micro BCA Protein Assay Kit (Thermo Fisher Scientific). 200 ng of each sample were analyzed by LC–MS/MS with a nanoHPLC UHPLC system (Bruker Daltonik GmbH, Billerica, MA, USA) with an Aurora series reversed‐phase C18 column (25 cm × 75 μm i.d., 1.6 μm C18, IonOpticks) heated to 50°C and coupled to a TimsTOF Pro2 (Bruker Daltonik GmbH). A gradient of 2%–35% B, where mobile phase A was 0.1% formic acid in water and B was 0.1% formic acid in acetonitrile, was used for 1 h. The total run time, including a ramp up to 35%–95% B to clean the column, and prepare it for the next sample. The mass spectrometer was set to PASEF scan mode for DDA acquisition spanning 300–1250 m/z with 10 PASEF ramps. The TIMS settings were 100 ms ramp and accumulation time (100% duty cycle) and a ramp rate of 9.42 Hz; this resulted in 1.17 s of total cycle time. Linear precursor repetitions were set at a 20 000 target intensity with a 2500 intensity threshold. Active exclusion was enabled with a 0.4 min release. The collision energy remained at default with a base of 1.6 1/K0 [V s/cm^2^] set at 59 eV and 0.6 1/K0 [V s/cm^2^] at 20 eV. Isolation widths were set at 2 m/z at < 700 m/z and 3 m/z at > 800 m/z. TIMS ranges were set initially from one range of 0.6–1.6 1/K0 [V s/cm^2^] as seen in most published studies and further optimized and tested at a narrower range of 0.75–1.25 1/K0 [V s/cm^2^]. Mass spectrometry data were analyzed with Protein Pilot software (v4.5, Sciex) using the following parameters: (1) search against a database composed of *Homo sapiens* from SwissProt (release on September 2022, with 26 610 reviewed entries); (2) MMTS as fixed modification; (3) trypsin digestion (with a miss cleavage factor of 0.75, Paragon Algorithm). An independent False Discovery Rate (FDR) analysis using the target‐decoy approach provided by Protein Pilot was used to assess the quality of identifications. Positive identifications were considered when identified proteins and peptides reached a 5% local FDR.

### 2.12 Statistical Analysis

All statistical analyzes were realized using GraphPad Prism 7.02 (La Jolla, CA, USA). Normality of data distribution was assessed with the Shapiro–Wilk test (for the normal Gaussian distribution test), allowing for the choice of parametric or nonparametric statistical tests to analyze significance. Nonparametric unpaired *t*‐test Mann–Whitney or one‐sample t test and Wilcoxon test were used for comparison of two groups. Parametric or nonparametric one‐way ANOVA was used for comparison of more than two groups. A difference with *p* value < 0.05 was considered statistically significant (ns, nonsignificant, *p < 0.05, **p < 0.01, ***p < 0.001, ****p < 0.0001). Data are mean ± SEM for at least three independent experiments.

## 3 Results

### 3.1 Effects of LSS on Cell Alignment and Elongation in TeloHAEC and HUVEC

**FIGURE 1:**
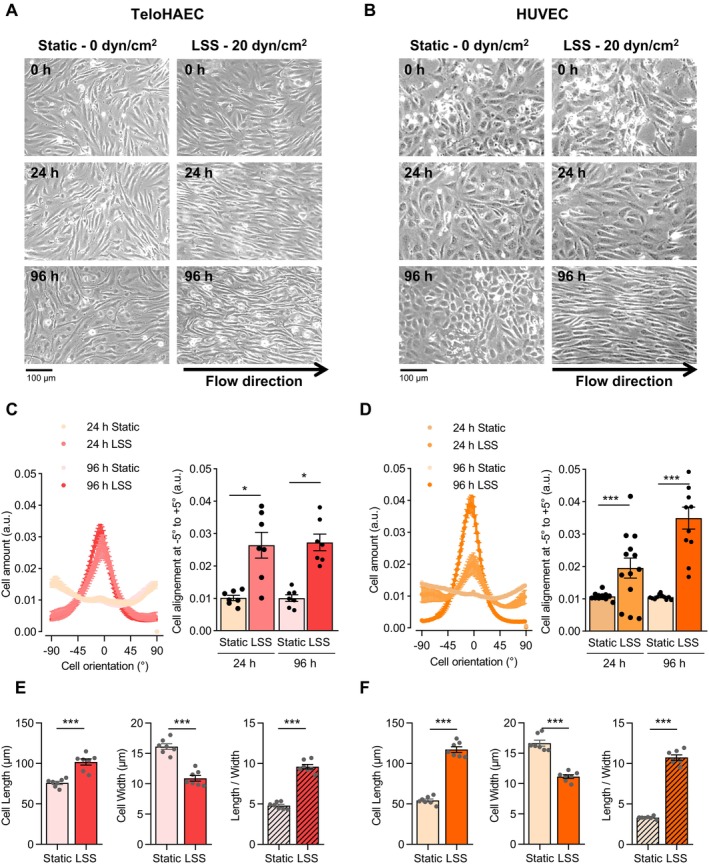
Phenotypic changes induced by LSS in TeloHAEC and HUVEC. Representative phase contrast microscopy images of TeloHAEC (A) and HUVEC (B) cultivated under static condition (Static, 0 dyn/cm^2^) or LSS (20 dyn/cm^2^) at different times (0, 24, and 96 h). TeloHAEC (C) and HUVEC (D) alignment quantification using local gradient orientation method with Fiji software at 24 and 96 h of LSS or under static condition. Cell amount is expressed over cell orientation in degree. A peak at angle value of 0° corresponds to a cell alignment with the flow direction. The histogram illustrates cell amount between angle values of −5° and +5°. Phenotypic modifications of TeloHAEC (E) and HUVEC (F) were assessed with the cell length (left panel) and width (middle panel), giving the elongation factor (Length/Width, right panel) after 96 h of LSS or of static culture. Values are mean ± SEM are shown (*n* = 7–13). Significance was analyzed using nonparametric Kruskal–Wallis (C, D) or Mann–Whitney (E, F) tests. *p < 0.05, ***p < 0.001.

In order to investigate the role of Na_V_ channels in LSS endothelial response, we used the TeloHAEC, which are a relevant model for in vitro study of LSS response as they originate from human aorta [32, 33]. Since there are no previous LSS experiments described to date, we first characterized the behavior of these cells in static condition and upon flow (20 dyn/cm^2^) in comparison to the well‐known EC model, HUVEC [36] (Figure 1). As shown in cell images, TeloHAEC are mostly aligned and became more elongated in the flow direction after 24 and 96 h, as HUVEC (Figure 1A,B). The analysis of TeloHAEC phenotype under LSS showed a peak at 0°, reflecting that almost all cells exhibited an angle value of 0°, corresponding to cell alignment with the flow direction. In static condition, TeloHAEC did not show a particular distribution, rather a random orientation (Figure 1C). Histograms showed that the quantity of TeloHAEC aligned between the angle values of −5° to +5° was significantly higher under LSS condition than static conditions (*p* < 0.05 at 24 h and *p* < 0.01 at 96 h). HUVEC behaved similarly, as expected, but were less aligned than TeloHAEC after 24 h (Figure 1D). As such, we performed the next analysis after 96 h of LSS. The lengths of TeloHAEC significantly increased, from 75.9 ± 1.9 μm to 101.7 ± 3.8 μm after 96 h of LSS (*p* < 0.001), whereas their widths decreased from 16.1 ± 0.5 μm to 10.9 ± 0.5 μm (*p* < 0.001) (Figure 1E). As a consequence, the cell elongation factor (ratio of cell length/cell width) was significantly higher in TeloHAEC by 2‐fold from 4.8 ± 0.16 μm to 9.6 ± 0.3 μm (*p* < 0.001) (Figure 1E). Similar observations were made with HUVEC under LSS (Figure 1F). Taken together, these data showed that 96 h‐LSS exposure at 20 dyn/cm^2^ induced similar phenotypic modifications in both HUVEC and TeloHAEC, as cell alignment and elongation along the flow direction.

### 3.2 Effects of LSS on Signaling Pathways in TeloHAEC and HUVEC

**FIGURE 2:**
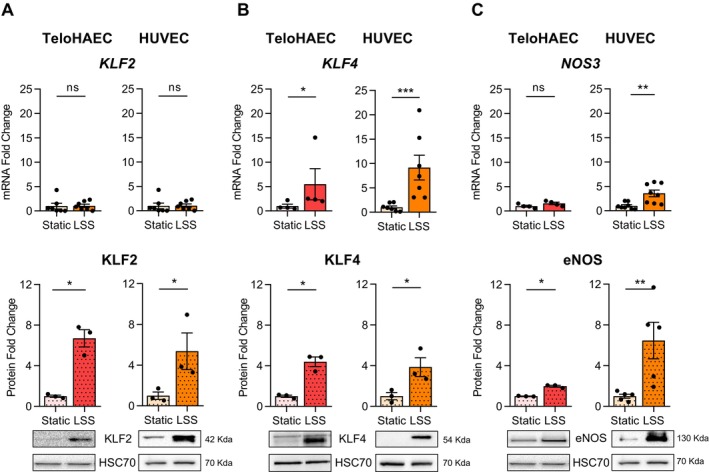
Activation of the atheroprotective signaling pathway in TeloHAEC and HUVEC by LSS. The transcriptional and protein modification of *KLF2*/KLF2, *KLF4*/KLF4, and NOS3/*eNOS* expression induced by LSS (20 dyn/cm^2^, 96 h) was measured in TeloHAEC and HUVEC, by RT‐qPCR and western blotting, followed by densitometry analysis (A–C). The histogram bars illustrate the fold change values (mRNA and protein) as mean ± SEM (*n* = 3–8), compared to static condition, given the arbitrary value of 1 for RT‐qPCR data or normalized to HSC70 (loading control protein) and compared to static condition, given the arbitrary value of 1 for western blotting. Nonparametric Mann–Whitney tests were performed. ns, nonsignificant, *p < 0.05, **p < 0.01, ***p < 0.001.

Next, we examined whether atheroprotective LSS (20 dyn/cm^2^, 96 h) modified the expression of KLF2, KLF4, and eNOS at mRNA and protein levels, in TeloHAEC, as in HUVEC (Figure 2). While no change in *KLF2* mRNA expression was observed in TeloHAEC (*p* = 0.68), KLF2 protein expression was significantly increased by LSS with fold change values of 6.7 ± 0.85 (*p* < 0.05) (Figure 2A). The expression of KLF4 was increased at the levels of mRNA with a fold change of 5.5 ± 3.1 (*p* < 0.05) and also at the protein level with a fold change of 5.5 ± 3.1 (*p* < 0.05) (Figure 2B). No change in *NOS3* mRNA expression was found (*p* = 0.06), while a significant increase in protein expression (fold change values of 3.6 ± 0.7, *p* < 0.01) occurred in TeloHAEC (Figure 2C). Similar observations were made in HUVEC (Figure 2A–C, right panel). Thus, the atheroprotective signaling pathway (KLF2/KLF4/eNOS) is efficiently activated by LSS in TeloHAEC, as in HUVEC.

### 3.3 Effects of LSS on the Expression of Na_V_ Channel Subunits in TeloHAEC and HUVEC

**FIGURE 3:**
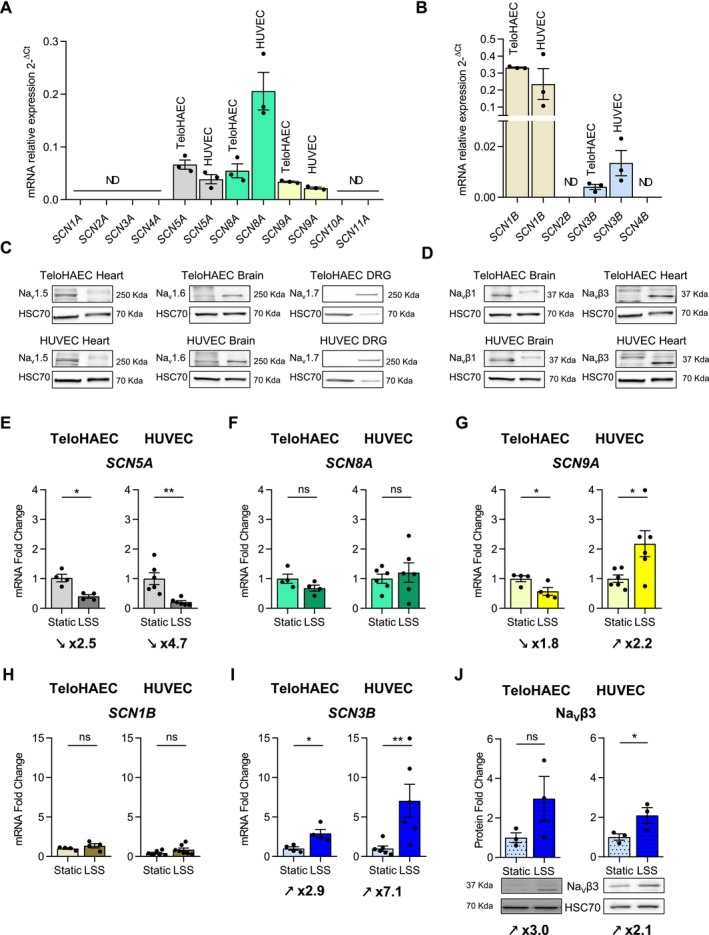
LSS modulates Na_V_ expression profiles in both TeloHAEC and HUVEC after 96 h of flow. mRNA expression profile of Na_V_ channel subunits (*SCN1A* to *SCN11A* and *SCN1B* to *SCN4B*) in TeloHAEC and HUVEC (A, B). The mRNA relative expression of Na_V_ channel subunits was determined by relative RT‐qPCR. The data are mean of mRNA relative expression ± SEM (*n* = 3) with the 2^−ΔCq^ method. ND, not detected (Cq > 35). The protein expression of Na_V_ channel subunits detected at transcript level was evaluated by western blot in comparison to control tissues as mouse heart (for Na_V_1.5 and Na_V_β3), brain (for Na_V_1.6 and Na_V_β1) or Dorsal Root Ganglion neurons (DRG) (for Na_V_1.7) (C, D). Fifty μg of cell protein extracts (TeloHAEC and HUVEC) and twenty μg of protein from control tissue (heart, brain and DRG) were loaded on 8% SDS‐PAGE. HSC70 protein was used as a loading control (C, D). In TeloHAEC or HUVEC, Na_V_1.5 and Na_V_β3 share the same HSC70 loading control as well as Na_V_1.6 and Na_V_β1 since the protein samples immunoblotted with these antibodies (Na_V_1.5/Na_V_β3 and Na_V_1.6/Na_V_β1) are the same after cutting the nitrocellulose membrane. *SCN5A* (E), *SCN8A* (F) and *SCN9A* (G), *SCN1B* (H), *SCN3B* (I) mRNA expression and Na_V_β3 protein expression (J) in LSS (20 dyn/cm^2^ for 96 h) compared to static condition in TeloHAEC (left panel) and HUVEC (right panel). Values of fold change (mRNA and protein) are expressed as mean ± SEM for at least 3 until 6 independent experiments and compared to static condition, given the arbitrary value of 1 using the 2−^ΔΔCq^ method for RT‐qPCR experiments or normalized to HSC70 used as loading control protein and compared to static condition, given the arbitrary value of 1 for the densitometry analysis. Immunoblot representative images of Na_V_β3 and HSC70 protein expression in TeloHAEC and HUVEC (J) cultivated under static or LSS (20 dyn/cm^2^) condition for 96 h. Nonparametric Mann–Whitney tests were performed. ns, nonsignificant, *p < 0.05, **p < 0.01.

To investigate whether Na_V_ channels contribute to mechanosensitive responses, we first characterized their expression in TeloHAEC and HUVEC in static and LSS conditions (Figure 3). *SCN5A*, *SCN8A*, and *SCN9A* transcripts were detected in both TeloHAEC and HUVEC in static condition (Cq values ~30 for 10 ng of cDNA except for *SCN8A* in HUVEC which give at Cq value of ~27) (Figure 3A). In contrast, *SCN1A*, *SCN2A*, *SCN3A*, *SCN4A*, *SCN10A*, and *SCN11A* transcripts were amplified at very low levels (Cq > 35 for 10 ng of cDNA), indicating that these genes were not expressed in TeloHAEC and HUVEC (Figure 3A). In these EC types, *SCN1B* and *SCN3B* cDNAs were detected with Cq values of ~27 and ~32 for 10 ng of cDNA respectively, while no expression was observed for *SCN2B* and *SCN4B* (Figure 3B). The expression of the corresponding Na_V_ channel subunits was validated at protein levels, that is, Na_V_1.5, Na_V_1.6, Na_V_β1, and Na_V_β3 types, except for Na_V_1.7 (Figure 3C,D).

We next studied the impact of LSS on *SCN5A*, *SCN8A*, *SCN9A, SCN1B*, and *SCN3B* mRNA levels expressed endogenously in TeloHAEC and HUVEC. A significant downregulation of *SCN5A* expression was observed after 96 h of LSS with a 2.5‐fold change in TeloHAEC (*p* < 0.05) and a 4.7‐fold change in HUVEC (*p* < 0.01) (Figure 3E). The expression level of *SCN8A* mRNA did not change in TeloHAEC (*p* = 0.1) and also in HUVEC (*p* = 0.94) (Figure 3F). Surprisingly, the expression of *SCN9A* decreased in TeloHAEC (fold change of 0.6, *p* < 0.05) and increased in HUVEC (fold change of 2.2, *p* < 0.05) (Figure 3G). *SCN1B* expression did not significantly change in TeloHAEC (*p* = 0.17) and HUVEC (*p* = 0.19) (Figure 3H). Even more surprisingly, *SCN3B* expression level was increased by LSS by a 2.9‐fold in TeloHAEC (*p* < 0.05) and a 7.1‐fold in HUVEC (*p* < 0.01) (Figure 3I). At the protein level, we performed a western blot analysis only for Na_V_1.5 and Na_V_β3, which showed the strongest variation in mRNA expression in the same direction. However, no change in Na_V_1.5 protein level was observed after LSS in both EC types (Table S2). Concerning Na_V_β3, the 3‐fold increase in protein expression level was found not significant in TeloHAEC (*p* = 0.1), while a significant 2.1‐fold increase (*p* < 0.05) was measured in HUVEC (Figure 3J). We assumed that the Na_V_β3 expression variation in TeloHAEC was not significant due to the weak expression of this protein in static conditions, as shown by the western blot (Figure 3J).

Taken together, the expression profile of Na_V_ channels is similar in TeloHAEC and HUVEC, at transcript (*SCN5A*, *SCN8A*, *SCN9A*, *SCN1B*, and *SCN3B*) and protein levels (Na_V_1.5, Na_V_1.6, Na_V_β1, and Na_V_β3), in static condition. Our data also show that LSS modulates Na_V_1.5 and Na_V_β3 subunit expression. Na_V_1.5 is downregulated by LSS at the transcript level in both TeloHAEC and HUVEC, but we were not able to validate this observation at the protein level. Interestingly, Na_V_β3 expression was strongly upregulated by LSS in TeloHAEC and HUVEC. Thus, we focused our investigation on Na_V_β3 and its contribution in the endothelial mechanosignaling.

### 3.4 Involvement of KLF4 Transcription Factor in SCN3B Expression Under Static and LSS Conditions in TeloHAEC

**FIGURE 4:**
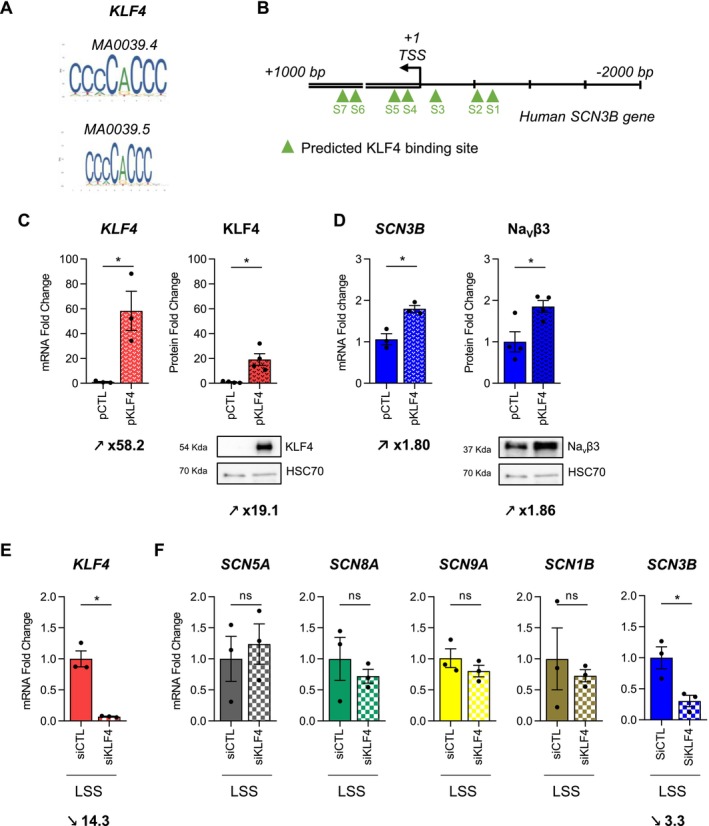
KLF4 is involved in *SCN3B* mRNA and protein expression in TeloHAEC. (A) Graphical representation of the KLF4 based on the position frequency matrix (PFM). The height of the column at each position is the information content (bit) and the individual base heights are in proportion to their frequencies. (B) Schematic representation of the location of the seven putative KLF4 (green triangles) DNA binding sites in human *SCN3B* promoter, with a score greater than 10.00, given by JASPAR analysis, named S1 to S7. (C, D) The effects of KLF4 overexpression using pKLF4 in comparison with empty vector (pCTL) were evaluated on KLF4 mRNA and protein (C) and *SCN3B* mRNA and Na_V_β3 protein (D) expression in TeloHAEC. (E, F) The effects of LSS (24 h at 20 dyn/cm^2^) on *KLF4* (E) and *SCN5A*, *SCN8A*, *SCN9A*, *SCN1B*, and *SCN3B* (F) expression were evaluated in TeloHAEC transfected with SiRNA Control (SiCTL) or SiRNA targeting KLF4 (SiKLF4). The histogram bars illustrate the fold change values (mRNA and protein) as mean ± SEM (*n* = 3–4), compared with pCTL or SiCTL, given the arbitrary value of 1 for RT‐qPCR data using the 2^−ΔΔCq^ method or normalized to HSC70 (loading control protein) and compared to pCTL, given the arbitrary value of 1 for western blotting. Values of mRNA fold change are expressed as mean ± SEM (*n* = 3) and compared to SiCTL condition, given the arbitrary value of 1. Significance was analyzed using nonparametric Mann–Whitney tests. ns, nonsignificant, *p < 0.05.

In order to understand how Na_V_β3 expression was increased by LSS in both EC types, we focused on one of the main signaling pathways activated by LSS, that is, KLF2/KLF4 transcription factors (Figure 4A). Since KLF4 acts upstream by regulating the expression of KLF2 [37], we first examined whether the *SCN3B* promoter contains a consensus‐binding element for KLF4 respectively named MA0039.4 and MA0039.5 (Figure 4A). Among the 7 putative binding sites for KLF4 called S1 to S7 present in the Human *SCN3B* gene, the Jaspar analysis highlighted 4 putative binding sites for KLF4 before the transcription start site (TSS +1), thus belonging to the promoter region of *SCN3B* (Figure 4B and Table S3). To investigate the involvement of KLF4 in the regulation of *SCN3B* expression, TeloHAEC were transfected with pKLF4 and with the control vector (pCTL) (Figure 4C,D). After 24 h, a significant increase in KLF4 expression was observed with a fold change value of 58.2‐fold for mRNA and 19.1‐fold for protein levels (*p* < 0.05) (Figure 4C). Interestingly, this KLF4 overexpression was associated with a significant increase in *SCN3B* mRNA level (1.80‐fold, *p* < 0.05) and Na_V_β3 protein level (1.86‐fold, *p* < 0.05) (Figure 4D). Next, we decided to characterize the effects of KLF4 silencing using SiRNA on *SCN5A*, *SCN8A*, *SCN9A*, *SCN1B*, and *SCN3B* in TeloHAEC after LSS. The SiRNA targeting *KLF4* (siKLF4) efficiently and strongly repressed *KLF4* expression in TeloHAEC after LSS with a fold change value of 14.3‐fold (*p* < 0.05) (Figure 4E). While siKLF4 did not impact *SCN5A*, *SCN8A*, *SCN9A*, and *SCN1B* expression, it induced significantly a repression of *SCN3B* expression in TeloHAEC after LSS with a fold change value decrease of 3.3 (*p* < 0.05) (Figure 4F). Taken together, these data evidence that KLF4 induction is involved in Na_V_β3 expression under static conditions and triggered *SCN3B* expression in EC under LSS.

### 3.5 Effect of SiRNA Targeting SCN3B on Alignment and Morphology of TeloHAEC Under LSS

**FIGURE 5:**
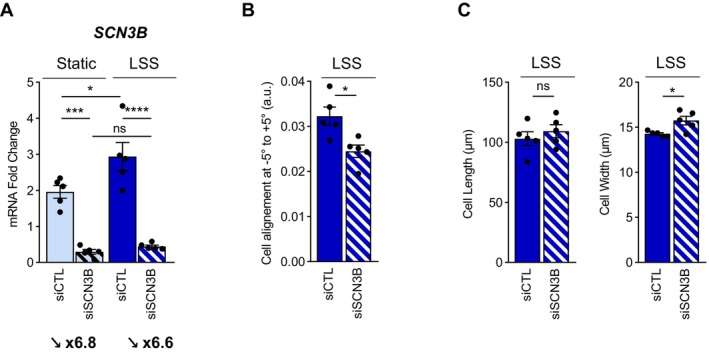
SiRNA *SCN3B* decreased cell alignment and increased cell width after 24 h of LSS in TeloHAEC. (A) mRNA expression of *SCN3B* in TeloHAEC transfected with SiRNA Control (siRNA CTL) or SiRNA targeting *SCN3B* (SiRNA *SCN3B*) by ddPCR. Values of *SCN3B* mRNA fold change are expressed as mean of copy number of *SCN3B* per μL. (B) The histogram illustrates TeloHAEC amount between angle values of −5° and +5°, after transfection with SiRNA Control (siRNA CTL) or SiRNA targeting *SCN3B* (siRNA *SCN3B*). (C) Elongation of HUVEC was determined as the cell length along flow direction (left panel) divided by cell width (right panel). TeloHAEC were submitted to static or LSS (20 dyn/cm^2^) for 24 h. Values are mean ± SEM are shown (*n* = 5). Significance was analyzed using one‐way ANOVA statistical analysis, followed by Tukey multiple comparisons test or nonparametric Mann–Whitney tests. ns, nonsignificant, *p < 0.05, ***p < 0.001, ****p < 0.0001.

To establish the link between *SCN3B* and endothelial response to LSS, TeloHAEC were transfected with SiRNA targeting *SCN3B* (SiSCN3B) prior to static or LSS conditions for phenotype characterization (Figure 5). Since the expression level of *SCN3B* was very low in conventional RT‐qPCR (Cq values of ~32 for 10 ng of cDNA), we evaluated *SCN3B* expression using ddPCR technology, which is much more sensitive [38]. While *SCN3B* expression was significantly induced by LSS at 24 h with a fold change value of 1.5 (*p* < 0.05), the SiSCN3B efficiently decreased SCN3B expression significantly with a fold change of ~6.7 in both static (*p* < 0.001) and LSS (*p* < 0.0001) conditions (Figure 5A). The quantification of TeloHAEC alignment showed that SiSCN3B significantly decreased cell alignment in comparison with SiCTL condition under LSS (*p* < 0.05) (Figure 5B). Moreover, the cell dimension analysis showed that TeloHAEC width was significantly increased with SiSCN3B (*p* < 0.05) (Figure 5C). Thus, altogether these data show that *SCN3B* expression is correlated to the alignment and morphology of TeloHAEC after LSS.

### 3.6 Identification of Protein Partners of Na_V_β3 by Proteomic Analysis

**FIGURE 6:**
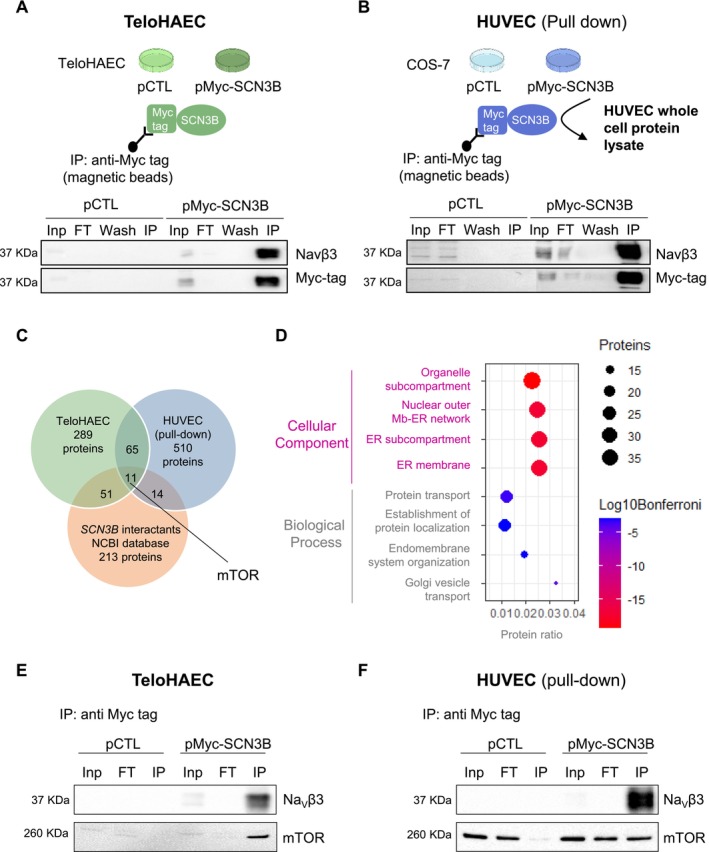
Identification of Na_V_β3‐interacting partners in TeloHAEC and HUVEC by proteomic analysis. (A, B) Schematic representation of the Na_V_β3 immunoprecipitation (IP) procedures. TeloHAEC (A) and COS‐7 (B) were transfected with pMyc‐SCN3B, and pCTL (negative control). (A) Myc tag‐Na_V_β3 with its partners were coimmunoprecipitated using anti‐Myc tag magnetic beads from TeloHAEC protein extracts. (B) Myc tag‐Na_V_β3 from COS‐7 protein extracts were first immunocaptured with anti‐Myc tag magnetic beads and subjected to pull‐down using HUVEC whole cell protein lysate. Below the schemes are shown representative control immunoblots (*n* = 3) with anti‐Na_V_β3 (top blot) and anti‐myc tag (bottom blot). Input (Inp) was the whole protein extract after cell transfection and the flow throw (FT) corresponded to the supernatant recuperated after immunocapturing with the anti‐Myc tag magnetic beads. Wash and IP correspond to the washing step and a sample from the solution used to boil the beads (IP) to elute Na_V_β3 and its partners. IP from TeloHAEC and HUVEC were subjected to MS/MS analysis. (C) Venn Diagram indicating the number of Na_V_β3 interacting proteins identified in TeloHAEC and HUVEC after proteomic analysis. (D) Bubble plots of Gene ontology (GO)‐term enrichment analysis of the 76 putative Na_V_β3 interactants. The four first GO‐terms belonging to the biological process and cellular component categories are indicated on the left. Below the bubble chart, is shown the protein ratio (number of proteins in our dataset compared to number of proteins in annotation). The scale of the bubble plot size (black, upper right) reflects the number of proteins. The significancy of the identification score is illustrated by a color intensity scale (Log10 Bonferroni, lower right). (E, F) Representative Na_V_β3 and mTOR immunoblots (*n* = 3) of immunoprecipitated Na_V_β3 with Myc tag antibody in TeloHAEC (E) and HUVEC (F).

Next, we aimed to identify Na_V_β3 protein partners in EC by two distinct immunoprecipitation strategies using TeloHAEC and HUVEC (Figure 6). Thus, following transfection with the pMyc‐SCN3B vector to express Myc‐Na_V_β3 in TeloHAEC (Figure 6A), the coimmunoprecipitated Myc‐Navβ3 proteins by anti‐Myc‐Trap magnetic agarose beads in TeloHAEC were directly subjected to LC–MS/MS and proteomic analysis (Figure 6A). COS‐7 cells were also transfected with the pMyc‐SCN3B vector, and anti‐Myc‐Trap magnetic agarose beads were used to immunocapture Myc‐Navβ3, followed by a pull‐down assay using a whole HUVEC protein lysate (Figure 6B). Then, the coimmunoprecipitated Myc‐Navβ3 proteins were submitted to LC–MS/MS. Analysis with the DDA approach allowed the proteomic identification of 289 candidates as Na_V_β3 partners in TeloHAEC and 510 in HUVEC (Figure 6C and Table S4). Our data allowed the identification of 65 novel *SCN3B* interactants, shared by TeloHAEC and HUVEC. 14 candidates in TeloHAEC and not in HUVEC are included in the *SCN3B* interactants list given by the GenBank database. 50 candidates in HUVEC and not in TeloHAEC are present in the GenBank *SCN3B* interactants list. Finally, 11 candidates identified in both TeloHAEC and HUVEC are also in the GenBank *SCN3B* interactants list (Figure 6C). A bioinformatic analysis of the functional roles was performed only on the 76 candidates shared in TeloHAEC and HUVEC (Table S4), using a gene ontology (GO)‐term enrichment analysis using the ToppGene database. We focused on 4 GO‐terms of two categories, that is, cellular component and biological process. Concerning the cellular component category, 30–40 proteins were distributed in the following GO‐terms: organelle subcompartment, nuclear outer membrane–endoplasmic reticulum (ER) network, ER subcompartment, and ER membrane. Concerning the biological process category, 10–20 proteins are connected to the following functions: protein transport, establishment of protein localization, endomembrane system organization, and Golgi vesicle transport (Figure 6D).

Several interesting outputs of this analysis can be highlighted, but we choose to focus on mTOR, included in the 11 candidates shared by both EC type and the GenBank *SCN3B* interactants list, because mTOR is present in all cellular component GO‐terms and associated with autophagy, which is necessary for EC alignment under shear stress [15].

Thus, we decided to validate the interaction between Na_V_β3 and mTOR in EC by proceeding as described in Figure 6A,B, and western blotting with mTOR antibody. The immunoprecipitation of Myc tag‐Na_V_β3 in TeloHAEC allowed the immunodetection of mTOR (Figure 6E). The Myc tag‐Na_V_β3 expressed in COS‐7 also immunoprecipitated mTOR from whole HUVEC protein lysate (Figure 6F). mTOR was detected only in cells transfected with pMyc‐Na_V_β3 in IP conditions and not in cells transfected with negative control plasmid (pCTL) in TeloHAEC (Figure 6E). In HUVEC, mTOR was found in input (Inp), flow through (FT) in both conditions but only in IP conditions in cells transfected with pMyc‐Na_V_β3 and not in cells transfected with negative control plasmid (pCTL) (Figure 6F).

As such, these data demonstrate that among the Na_V_β3 partners identified by the proteomic approach, mTOR interacts physically with Na_V_β3 in EC, suggesting the implication of Na_V_β3‐mTOR‐mediated autophagy signaling.

### 3.7 Effect of SCN3B SiRNA on Autophagy Signaling Pathway in TeloHAEC Under LSS

**FIGURE 7:**
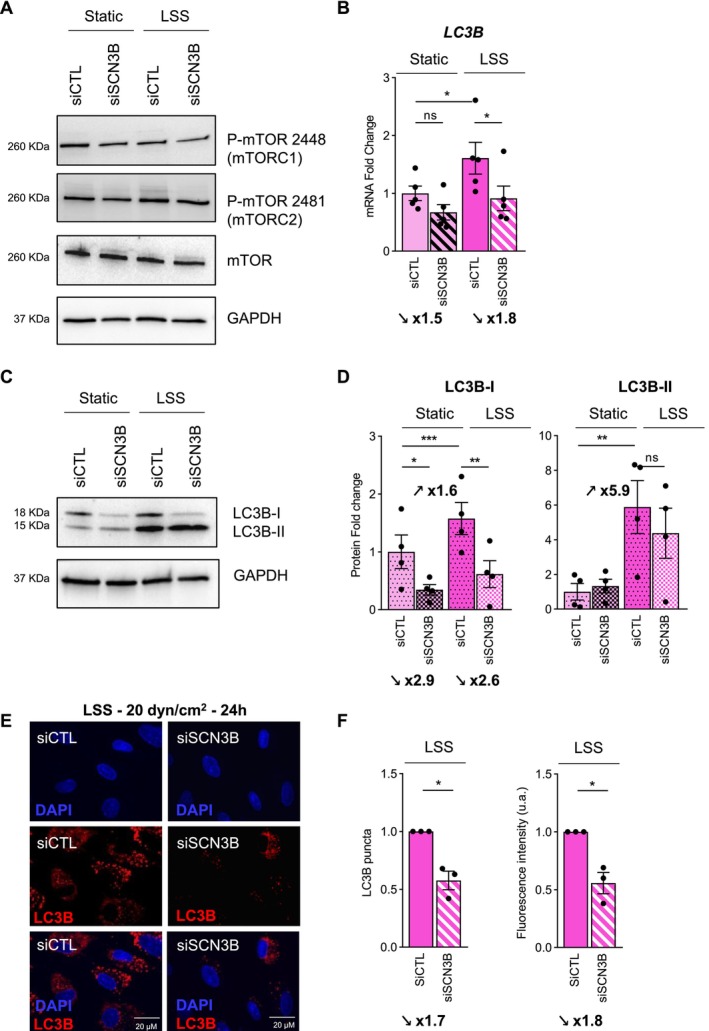
Na_V_β3 modulates LC3B expression in TeloHAEC. (A) Protein expression of P‐mTOR at serine 2448 (mTORC1 complex) or at serine 2481 (mTORC2 complex) and mTOR in static or LSS (20 dyn/cm^2^–24 h) conditions in TeloHAEC transfected with SiRNA Control (siCTL) or SiRNA targeting SCN3B (siSCN3B). The expression of proteins was evaluated by western blot where ten μg of cell protein extracts were loaded on 4%–20% SDS‐PAGE and where GAPDH protein was used as a loading control. (B) mRNA expression of *LC3B* in the same conditions. (C) LC3B protein expression in static or LSS (20 dyn/cm^2^–24 h) conditions in TeloHAEC transfected with SiRNA Control (siCTL) or SiRNA targeting SCN3B (siSCN3B). (D) Densitometry analysis following western blot of the LC3B‐I and LC3B‐II bands in static and LSS with or without siSCN3B in TeloHAEC. Values of fold change (mRNA and protein) are expressed as mean ± SEM for at least 4 or 5 independent experiments and compared to static condition, given the arbitrary value of 1 using the 2^−ΔΔCq^ method for RT‐qPCR experiments or normalized to GAPDH for western blot experiments. One‐way ANOVA was performed. ns, nonsignificant, *p < 0.05, **p < 0.01, ***p < 0.001. (E) Representative immunofluorescence images of LC3B staining (red) in TeloHAEC transfected with siCTL or siSCN3B and cultivated under LSS (20 dyn/cm^2^ for 24 h). Nuclei (blue) were stained with DAPI. (F) Histograms represent the number of puncta (left) and fluorescence intensity (right) of LC3B staining. Data are presented as mean ± SEM (*n* = 3) and normalized to SiCTL, given the arbitrary value of 1. Significance was analyzed using one sample *t*‐test and Wilcoxon test, *p < 0.05.

Since Na_V_β3 interacts with mTOR, a master negative regulator of autophagy, and because autophagy is a crucial component for cell alignment upon LSS and endothelial function [13, 14, 15, 16, 37], we then investigated the effects of SiSCN3B on the expression of the terminal effector of autophagy, LC3B, in TeloHAEC under static and LSS conditions. First, we observed that SiSCN3B did not modify the mTOR protein level nor the phosphorylation status of mTOR associated with mTORC1 or mTORC2 complexes in static and LSS conditions (Figure 7A). These results are in accordance with the observation that, despite other partners of the mTOR complex (mTORC) being detected by LC–MS/MS in the immunocaptured Myc‐Navβ3, these interactions have not been validated by subsequent western blot (data not shown). This concerned Raptor or mLST8 involved in the mTORC1 complex (Table S4) or Rictor involved in the mTORC2 complex.

Concerning LC3B expression under LSS and the impact of the SiRNA targeting *SCN3B* (Figure 7B), we found first that LSS led to a significant induction of *LC3B* mRNA with a fold change value of 1.6 in comparison with static condition (*p* < 0.05). Interestingly, SiSCN3B significantly decreased *LC3B* upregulation induced by LSS by 1.8‐fold (*p* < 0.05). Concerning LC3B protein levels, both LC3B‐I (cytosolic form) and LC3B‐II (membrane bound form present in autophagosomes) were significantly increased by LSS (Figure 7C) with fold change values of 1.6 ± 0.3 for LC3B‐I (*p* < 0.001) and 5.9 ± 1.5 (*p* < 0.01) for LC3B‐II (Figure 7D). Interestingly, SiSCN3B induced a significant 2.9‐fold and 2.6‐fold decrease in LC3B‐I protein expression respectively in both static and LSS conditions (*p* < 0.05, *p* < 0.01, respectively) (Figure 7C,D). However, SiSCN3B did not impact LC3B‐II protein expression (*p* = 0.99) where expression was strongly induced by LSS with a fold change value of 5.9 (Figure 7C,D).

Interestingly, the repression of *SCN3B* expression under LSS by SiRNA silencing drastically dropped the number of autophagosomes characterized by LC3B puncta or dots present inside the cells as well as LC3B fluorescence intensity, illustrated by immunofluorescence images of LC3B staining (Figure 7E). Indeed, the number of LC3B dots decreased by 1.7‐fold in SiSCN3B treated cells (*p* < 0.05) in comparison with SiCTL condition (Figure 7F, left panel) with a mean of 178 ± 43 puncta by field counted in SiCTL‐treated cells in comparison with 99 ± 26 puncta in SiSCN3B‐treated cells under LSS (Table S5). This diminution of puncta numbers was correlated to LC3B fluorescence intensity which decreased by 1.8‐fold in SiSCN3B condition compared to SiCTL (*p* < 0.05) (Figure 7F, right panel). Taken together, our results show that SiRNA targeting *SCN3B* does not influence the protein level or phosphorylation status of mTOR but alters the increase of LC3B autophagosome puncta number and LC3B expression induced by LSS.

### 3.8 Colocalization of Na_V_β3 With LC3B, LAMP1 and mTOR in TeloHAEC Under Static and LSS Conditions

**FIGURE 8:**
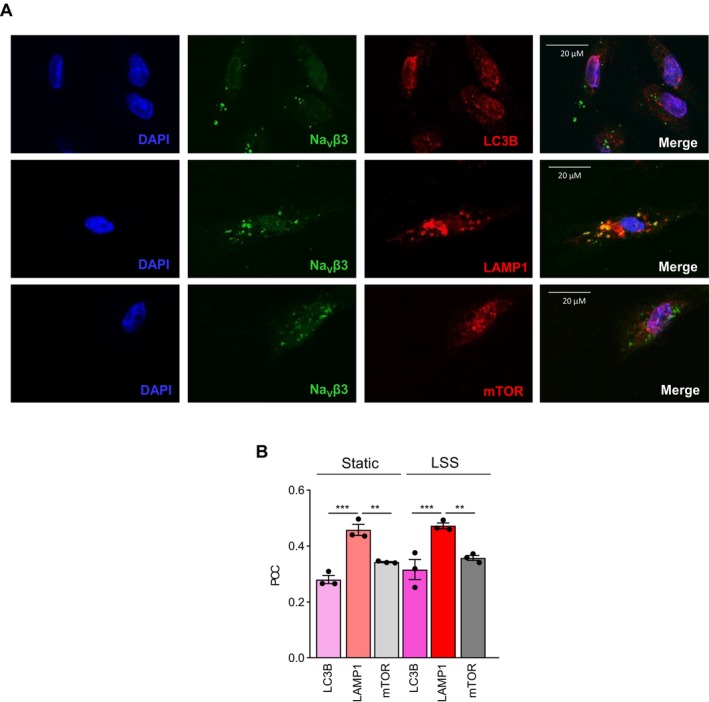
Na_V_β3 partially colocalizes with LC3B, LAMP1, and mTOR in TeloHAEC under static or LSS conditions. (A) Representative images illustrating the immunostaining of LC3B, LAMP1 or mTOR (red) together with Na_V_β3 (green) in TeloHAEC which were transfected with pmyc‐SCN3B and cultivated under static condition. Nuclei (blue) were stained with DAPI. (B) Quantification of the colocalization of Na_V_β3 with LC3B, LAMP1 or mTOR was calculated using Pearson's correlation coefficient (PCC). Histogram bars represent the PCC value as mean ± SEM for each protein (LC3B, LAMP1 or mTOR) in TeloHAEC cultivated under static or LSS conditions for three independent experiments. Significance was analyzed using one‐way ANOVA statistical analysis, followed by Tukey multiple comparisons test. **p < 0.01, ***p < 0.001.

Next, we investigated whether Na_V_β3 colocalizes with specific markers of organelles such as LC3B for autophagosomes or LAMP1 for late endosomes and lysosomes [40] (Figure 8). While a membrane distribution was expected, Na_V_β3 was immunolabeled in the nuclei and the cytosol. We did not observe clear Na_V_β3 immunolabeling at the plasma membrane, but rather a puncta labeling distribution (Figure 8). The immunofluorescence images illustrate a colocalization of Na_V_β3 with LAMP1, but not a clear colocalization with LC3B and mTOR (Figure 8A). Subsequent analysis of colocalization between Na_V_β3 and proteins of interest in TeloHAEC under static and LSS conditions was analyzed using the Pearson's correlation coefficient (PCC) which measures the pixel‐by‐pixel covariance in the signal levels of two images (Figure 8B). The colocalizations of Na_V_β3 with LC3B, LAMP1, and mTOR were evidenced by significant PCC values greater than 0 but below 1, encompassing between 0.28 and 0.47 (Table S6). Thus, we showed that Na_V_β3 colocalized with LAMP1 to a greater extent than LC3B, since PCC values for LAMP1 were significantly higher than those obtained for LC3B, with LAMP1 PCC values of 0.46 ± 0.02 under static condition (*p* < 0.001) and 0.47 ± 0.01 LSS condition (*p* < 0.001) (Figure 8B). No significant differences were observed between PCC values calculated for LC3B, LAMP1, and mTOR, respectively, in both static and LSS conditions. Altogether, our data show that the majority of Na_V_β3 is retained intracellularly, specifically within the late endosomes and lysosomes and to a lesser extent with autophagosomes, and interacts with mTOR similarly in static and LSS conditions.

### 3.9 Effects of Resveratrol on SCN3B Expression in HUVEC and TeloHAEC

**FIGURE 9:**
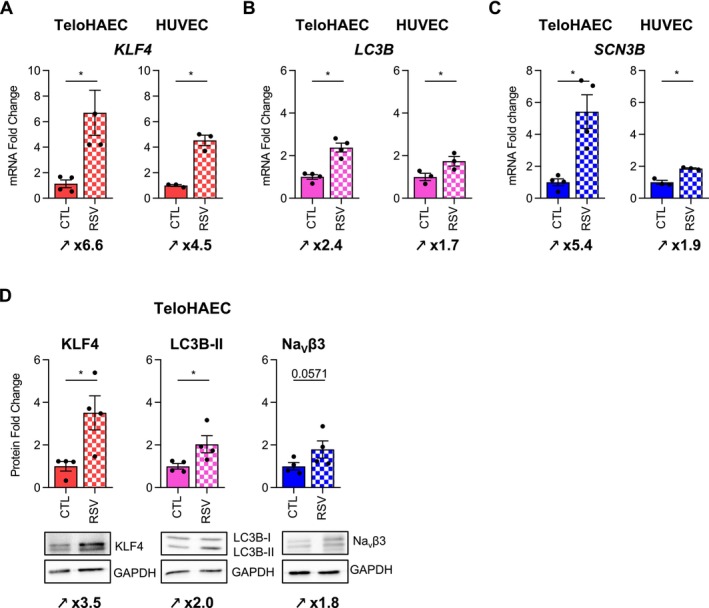
Resveratrol induces *KLF4*, *LC3B*, and *SCN3B* mRNA expression in both TeloHAEC and HUVEC and in KLF4, LC3B, and Na_V_β3 protein in TeloHAEC. (A‐C) The effects of RSV were evaluated on the expression of *KLF4* (A), *LC3B* (B), and *SCN3B* (C) mRNA in TeloHAEC and HUVEC. (D) The effect of resveratrol was assessed on KLF4, LC3B and Na_V_β3 protein expression in TeloHAEC. (A–D) The cells were treated with 100 μM of RSV and 0.1% DMSO (CTL) as negative control. The histogram bars illustrate the fold change values (mRNA and protein) as mean ± SEM (*n* = 3–4), compared to CTL, given the arbitrary value of 1 for RT‐qPCR data using the 2−^ΔΔCq^ method or normalized to GAPDH (loading control protein) and compared to CTL, given the arbitrary value of 1 for western blotting. Nonparametric unpaired Mann–Whitney tests were performed, *p < 0.05.

To support the idea that Na_V_β3 is involved in the autophagy process and vasculoprotection, EC were treated with resveratrol (RSV), which induces an atheroprotection [41] through the activation of KLF4 signaling [42] and autophagy [16, 17]. RSV exposition (100 μM for 48 h) led to an increase in KLF4 expression with a 6.6‐fold change in TeloHAEC (*p* < 0.05) and a 4.5‐fold change in HUVEC (*p* < 0.05) (Figure 9A) together with the autophagic marker *LC3B* expression with a 2.4‐fold change in TeloHAEC (*p* < 0.05) and a 1.7‐fold change in HUVEC (*p* < 0.05) (Figure 9B). Interestingly, *KLF4* and *LC3B* induction mediated by RSV was correlated with a strong increase in *SCN3B* expression in TeloHAEC (fold change value of 5.4, *p* < 0.05) and a moderate but significant induction in HUVEC (fold change value of 1.9, *p* < 0.05) (Figure 9C). Induction of *SCN3B* seems to be specific to this gene family since *SCN1B* expression was not modulated by RSV in TeloHAEC (data not shown). The increase in KLF4, LC3B, and *SCN3B* by RSV was also observed at the protein level in TeloHAEC, where RSV induced an increase in KLF4, LC3B‐II, and Na_V_β3 with fold change values of 3.5, 2.0, and 1.8, respectively (Figure 9D). Taken together, these data show that RSV‐induced vasculoprotection and autophagy is associated with the activation of Na_V_β3 expression.

## 4 Discussion

The main output of this study is that the auxiliary Na_V_ subunit, Na_V_β3 plays a role in cell alignment in response to physiological shear stress. Indeed, we showed that in TeloHAEC, Na_V_β3 is induced by KLF4, a key regulator of endothelial function and atheroprotective signaling pathway. Moreover, we demonstrated that Na_V_β3 is expressed intracellularly mainly inside lysosomes, contributes to the regulation of the autophagy pathway involved in cell alignment through its interaction with mTOR and the modulation of the number of autophagosomes formation. Thereby, Na_V_β3 is a novel actor in EC mechanosignaling in response to shear stress and might contribute to vasculoprotection.

Under a physiological LSS, EC change their phenotype, going from a so‐called cobblestone shape to a more elongated shape and alignment in the flow direction [10]. We first showed that the immortalized human aortic endothelial cells TeloHAEC behaved as the classical EC model HUVEC, showing elongation and alignment under LSS. These morphological modifications are accompanied by activation of atheroprotective signaling pathways, that is, KLF2/KLF4/NOS3 induction in TeloHAEC, as previously shown in HUVEC [5, 11]. We then used TeloHAEC since they originate from human aorta, a type of artery prone to atherosclerosis [34, 35]. Therefore, TeloHAEC represent an interesting new model for the investigation of endothelial signaling pathway induced by shear stress.

Among endothelial mechanosensitive ion channels, Na_V_ channels have emerged as interesting candidates since they are involved in flow‐mediated vasodilation in resistance arteries [18, 19, 20, 38]. In this study, we found that TeloHAEC endogenously express Na_V_1.5, Na_V_1.6, Na_V_β1 and Na_V_β3, similarly to HUVEC. These data are in agreement with Andrikopoulos' study [20] in which *SCN5A*, *SCN8A*, *SCN9A*, *SCN1B*, and *SCN3B* expression has been reported in HUVEC, at similar levels to our RT‐qPCR data obtained in TeloHAEC. The expression of Na_V_ channel subunits in HUVEC has raised the idea of their noncanonical roles, that is, nonelectrogenic function, such as their contributions to the response of EC to shear stress [21].

We then found that LSS downregulates *SCN5A* expression. This downregulation could be due to the activation of the transcription factor FoxO1, known to be activated by LSS [39] and to directly repress *SCN5A* transcription. In HUVEC, the inhibition of Na_V_1.5 activity has been shown to decrease cell proliferation [18]; as such, the repression of *SCN5A* expression by LSS might be associated with the reduction in EC proliferation, which occurs under LSS [11].

Our main findings underlying that LSS induces Na_V_β3 are in agreement with a previous report in GSE datasets, showing that high shear stress leads to *SCN3B* mRNA induction (GSE23289) [44]. Moreover, our data show that the increase of *SCN3B* transcription is mediated by KLF4 activation since KLF4 overexpression induces Na_V_β3 expression, while the repression of KLF4 expression decreases *SCN3B* expression under flow. This finding is in accordance with transcriptomic analysis of HUVEC overexpressing KLF4 (GSE90982), showing that KLF4 induces an upregulation of *SCN3B* expression [45]. The induction of *SCN3B* could also be mediated by KLF2. Indeed, KLF4 and KLF2 share many target genes in common, such as *NOS3* [46] due to the similarity of the consensus sequences between KLF4 and KLF2. However, the prediction of response elements for KLF4 in the *SCN3B* gene exhibits much higher scores than those for KLF2. Furthermore, *SCN3B* mRNA is not upregulated in HUVEC infected with KLF2 lentivirus (GSE19412) [9] nor with KLF2 adenovirus [6]. In addition, other transcription factors might be involved in *SCN3B* upregulation under shear stress, such as FoxO1 [47] and p53 transcription factors [48]. Interestingly, both transcription factors have been highlighted as key activators of atheroprotective signaling pathways, as KLF4 [11].

Our data show that *SCN3B* downregulation impairs EC elongation and alignment in response to LSS in TeloHAEC. Other proteins belonging to the Ig‐CAM family, including Na_V_β3, such as VE‐Cadherin or PECAM1 (platelet endothelial cell adhesion molecule), have also been shown to be involved in EC alignment under flow [49, 50]. Additionally, the identification of the protein partners using proteomic analysis demonstrated that Na_V_β3 is a partner of mTOR, a negative regulator of autophagy, particularly upon cellular stress such as serum starvation [51]. Of note, autophagy is essential to EC alignment [13, 14, 15, 16, 37]. mTOR protein or its phosphorylation is not modulated by LSS, since LSS does not directly modulate or block the mTOR signaling pathway [37]. Thus, we assume that the binding of mTOR to Na_V_β3 leads to its sequestration and, in turn, allows the activation of autophagy by increasing autophagosome formation and LC3B expression and subsequently EC alignment. Nevertheless, it remains to determine how Na_V_β3 interacts with mTOR. It has been shown that mTOR kinase activity can be inhibited after direct interaction with FKBP12 (FK506‐binding protein 12) [51]. This result led us to hypothesize that Na_V_β3 could bind mTOR to the same site as FKBP12, leading to the inhibition of mTOR activity. Interestingly, we found that Na_V_β3 partially colocalized with mTOR, in accordance with our co‐coimmunoprecipitation results and the idea that Na_V_β3 could inhibit mTOR activity by direct interaction and subsequently increase the autophagic flux.

As an auxiliary subunit of Na_V_ channels belonging to the Ig‐CAM family, Na_V_β3 is expected to be inserted into the plasma membrane [52]. However, we demonstrated that Na_V_β3 is retained in subcellular compartments, mainly in lysosomes and to a lesser extent in autophagosomes and bound mTOR, which is known to be localized to the lysosomes [53]. The localization of Na_V_β3 is consistent with recent results concerning another Na_V_ channel β‐subunit isoform, Na_V_β1, which is expressed in the endoplasmic reticulum and endolysosomal vesicles [54]. Finally, the gene ontology‐term enrichment analysis highlights that several Na_V_β3 interactants belong to (i) cellular component of organelle compartment and (ii) biological process related to endomembrane system organization including TMED9 or EI24, both transmembrane proteins associated with autophagy [55, 56], emphasizing the new concept that Na_V_β3 is expressed in organelles and particularly in vesicles. Taken together, we propose Na_V_β3, expressed at the level of lysosomes, autophagosomes, or autolysosomes, as a novel actor of endothelial autophagy that blocks mTOR function through direct interaction.

Our data highlight the role of Na_V_ channels in the mechanosignaling pathway, induced by shear stress. In TeloHAEC and HUVEC, Na_V_1.5 and Na_V_β3 expressions are modulated by LSS, suggesting their contribution as mechanosensors. Indeed, it has been shown that Na_V_β1 and Na_V_β3 modulate the mechanosensitivity of Na_V_1.5 [28]. However, the fact that KLF4 silencing almost totally suppresses *SCN3B* induction in response to shear stress, suggest that Na_V_β3 is not directly a mechanosensor such as Piezo1 [57], but rather a downstream actor of the mechanosignaling pathway. Furthermore, it could be interesting to explore the relationship between Na_V_β3 and Na_V_1.5 in EC under LSS and the contribution of the resulting Na_V_ channel complex to membrane potential, Ca^2+^ homeostasis and endothelial function. Since *SCN3B* pathogenic variants are responsible of arrythmia [58], it would be very interesting to investigate their impact on shear stress response, and other endothelium function such as flow‐mediated dilation, that involves autophagic flux [18] or vasculoprotection since Na_V_β3 expression is increased by the atheroprotective resveratrol.

In conclusion, this study highlights a novel role of Na_V_β3 as a novel intracellular actor of endothelial mechanosignaling, involved in the autophagy pathway through mTOR interaction, which is crucial to cell alignment under shear stress and must contribute to vasculoprotection by increased autophagic flow.

## Author Contributions

Claire Legendre conceived and designed the research, performed the experiments, and acquired, analyzed, and interpreted the data, obtained grants, wrote, and edited the manuscript. Christian Legros conceived and designed the research, contributed to the discussion, and critically reviewed the manuscript. Léa Réthoré, Anne‐Laure Guihot, Linda Grimaud, Coralyne Proux, Alice Boissard, Cécile Henry, Jérôme Cayon, Rodolphe Perrot, Benjamin Barré, François Guillonneau, and Catherine Guette performed the experiments and acquired, analyzed, and interpreted the data. Daniel Henrion contributed to the discussion and reviewed the manuscript. All authors have approved the final version of the manuscript.

## Conflicts of Interest

The authors declare no conflicts of interest.

## Supporting information


Table S1.

Table S2.

Table S3.

Table S4.

Table S5.

Table S6.

## Data Availability

The data that support the findings of this study are available in the article and/or Supporting Information tables. Additional data supporting the findings of this study may be requested from the corresponding author.
