# Supplementary material for: A Novel Function of NaV Channel β3 Subunit in Endothelial Cell Alignment Through Autophagy Modulation
Source: FASEB J. 2025 May 30;39(11):e70663. doi: 10.1096/fj.202401558RR (PMC12124425; doi:10.1096/fj.202401558RR)
Supplement: Supplementary file 1 — Table S1. [file FSB2-39-e70663-s004.docx]

| **Gene name** | **GenBank accession number** | **Protein  name** | **Forward primer (5'-3')** | **Reverse primer (5'-3')** |
| --- | --- | --- | --- | --- |
| *SCN1A* | NM_001165963 | Na_V_1.1 | CCTACATCGCCTGTTGGAC | TCAGTTTCAGTGGTTGTTCCA |
| *SCN2A* | NM_001040142 | Na_V_1.2 | CCATGGAATTGGTTGGATTT | TGCTCGGAGAACTCTGAATG |
| *SCN3A* | NM_001081676 | Na_V_1.3 | TTTTACGTTTCTTCGTGATCCA | ATTGCCCAGGTCCACAAAC |
| *SCN4A* | NM_000334 | Na_V_1.4 | CAGCCTGAGGAGTGCTTCA | CGCAGAGTCCACCACTTCTT |
| *SCN5A* | NM_001160160 | Na_V_1.5 | CTGCACGCGTTCACTTTCCT | CAGCCAGCTTCTTCACAGACT |
| *SCN8A* | NM_001177984 | Na_V_1.6 | AGATCAGATGGAAGAACGTGAAG | ATGTCCATCCAGCCTTTGAA |
| *SCN9A* | NM_001365536 | Na_V_1.7 | CAACTTTTAAGGGATGGACGA | TCATATTTGGGCTGCTTGTCT |
| *SCN10A* | NM_001293306 | Na_V_1.8 | TCACGTACCTGAGAGATCCTTG | TCCCACGGAGATCTATTGCT |
| *SCN11A* | NM_001349253 | Na_V_1.9 | TCCTCCTTTTTGAGAGAGTCTGG | TTGGTTTGCTCTAGGAGCTGT |
|  |  |  |  |  |
| *SCN1B* | NM_001037 | Na_V_β1 | GGCACTGAGGAGTTTGTCAAG | GATCCTGCAGGTCTTTGGTG |
| *SCN2B* | NM_004588 | Na_V_β2 | ACGGGGCTCAGTCTCTTTTT | TCAGAGCCATTGAGGACGTT |
| *SCN3B* | NM_001040151 | Na_V_β3 | GATTGTTTCCCCTGGCTTCT | GTCTCCGACGGGCACTTCC |
| *SCN4B* | NM_001142348 | Na_V_β4 | GTCATCGGGCTCCTCATC | GGAGCTCACGAGACACTCCTT |
|  |  |  |  |  |
| *KLF2* | NM_016270 | KLF2 | AGGAGGTGACCAGTCTGGAA | AGGAGGTGACCAGTCTGGAA |
| *KLF4* | NM_001314052 | KLF4 | TTCCCATCTCAAGGCACACC | GCGAATTTCCATCCACAGCC |
| *KLF6* | NM_001300 | KLF6 | AAGCTCCCACTTGAAAGCACA | AAAACGCCACTCACACCCTT |
| *NOS3* | NM_000603 | eNOS | GACCCTCACCGCTACAACAT | CCGGGTATCCAGGTCCAT |
|  |  |  |  |  |
| *MAP1LC3B* | NM_022818 | LC3B | TCAGGTTCACAAAACCCGCC | GCGTTTGTGCCAACTGTGAT |
|  |  |  |  |  |
| *TBP* | NM_003194 | TBP | GAGCCAAGAGTGAAGA ACAGTC | GCTCCCCACCATATTCTGAATCT |
| *UBC* | NM_021009 | UBC | GGGATTTGGGTCGCAGTTCT | CACGAAGATCTGCATTGTCAAGT |
| *B2M* | NM_004048 | B2M | CTATCCAGCGTACTCCAAAGATT | TGGATGAAACCCAGACACATAG |
| **Supplementary Table S1. Primers used in this study** | | | |  |
