# Supplementary material for: A Novel Function of NaV Channel β3 Subunit in Endothelial Cell Alignment Through Autophagy Modulation
Source: FASEB J. 2025 May 30;39(11):e70663. doi: 10.1096/fj.202401558RR (PMC12124425; doi:10.1096/fj.202401558RR)
Supplement: Supplementary file 2 — Table S2. [file FSB2-39-e70663-s005.docx]

|  | **KLF2 protein (Figure 2)** | | |  |  |
| --- | --- | --- | --- | --- | --- |
|  | Conditions | Static | LSS |  |  |
|  | TeloHAEC | 1.00 ± 0.11 | 6.70 ± 0.85* |  |  |
|  | HUVEC | 1.00 ± 0.16 | 5.36 ± 1.8* |  |  |
|  |  |  |  |  |  |
|  | **KLF4 protein (Figure 2)** | | |  |  |
|  | Conditions | Static | LSS |  |  |
|  | TeloHAEC | 1.00 ± 0.12 | 4.4 ± 0.48* |  |  |
|  | HUVEC | 1.00 ± 0.35 | 3.86 ± 0.92* |  |  |
|  |  |  |  |  |  |
|  | **eNOS protein (Figure 2)** | | |  |  |
|  | Conditions | Static | LSS |  |  |
|  | TeloHAEC | 1.00 ± 0.02 | 1.99 ± 0.09* |  |  |
|  | HUVEC | 1.00 ± 0.19 | 6.47 ± 1.79** |  |  |
|  |  |  |  |  |  |

|  | **Nav1.5 protein (Figure 3)** | | |  |
| --- | --- | --- | --- | --- |
|  | Conditions | Static | LSS |  |
|  | TeloHAEC | 1.00 ± 0.23 | 1.43 ± 0.32 |  |
|  | HUVEC | 1.00 ± 0.19 | 1.15 ± 0.18 |  |
|  |  |  |  |  |
|  | **Navβ3 protein (Figure 3)** | | |  |
|  | Conditions | Static | LSS |  |
|  | TeloHAEC | 1.00 ± 0.25 | 2.98 ± 1.13 |  |
|  | HUVEC | 1.00 ± 0.16 | 2.09 ± 0.40* |  |
|  |  |  |  |  |

|  | **KLF4 protein (Figure 4)** | | |  |
| --- | --- | --- | --- | --- |
|  | Conditions | pCTL | pKLF4 |  |
|  | TeloHAEC | 1.00 ± 0.33 | 19.12 ± 4.67* |  |
|  |  |  |  |  |
|  |  |  |  |  |
|  | **Navβ3 protein (Figure 4)** | | |  |
|  | Conditions | pCTL | pKLF4 |  |
|  | TeloHAEC | 1.00 ± 0.24 | 1.85 ± 0.14* |  |
|  |  |  |  |  |

|  | **ratio P-mTOR2448/mTOR (Figure 7)** | | | | |  | |  |
| --- | --- | --- | --- | --- | --- | --- | --- | --- |
|  | Conditions | | Static | LSS | |  | |  |
|  | SiCTL | | 1.13 ± 0.31 | 1.30 ± 0.30 | |  | |  |
|  | SiSCN3B | | 1.12 ± 0.14 | 1.44 ± 0.27 | |  | |  |
|  |  | |  |  | |  | |  |
|  | **ratio P-mTOR2481/mTOR (Figure 7)** | | | | |  | |  |
|  | Conditions | | Static | LSS | |  | |  |
|  | SiCTL | | 1.31 ± 0.65 | 1.15 ± 0.59 | |  | |  |
|  | SiSCN3B | | 1.39 ± 0.53 | 1.24 ± 0.47 | |  | |  |
|  |  | |  |  | |  | |  |
|  | **LC3-I protein (Figure 7)** | | | | | |  | |
|  | Conditions | Static | | | LSS | |  | |
|  | SiCTL | 1.00 ± 0.29 | | | 1.58 ± 0.62*** | |  | |
|  | SiSCN3B | 0.34 ± 0.09* | | | 0.62 ± 0.23** | |  | |
|  |  | |  |  | |  | |  |
|  | **LC3-II protein (Figure 7)** | | | | |  | |  |
|  | Conditions | | Static | LSS | |  | |  |
|  | SiCTL | | 1.00 ± 0.48 | 5.88 ± 1.53** | |  | |  |
|  | SiSCN3B | | 1.33 ± 0.39 | 4.38 ± 1.45 | |  | |  |
|  |  | |  |  | |  | |  |

|  |  |  |  |  |
| --- | --- | --- | --- | --- |
|  | **LC3-I protein (Figure 9)** | | |  |
|  | Conditions | CTL | RSV |  |
|  | TeloHAEC | 1.00 ± 0.15 | 0.83 ± 0.32 |  |
|  |  |  |  |  |
|  | **LC3-II protein (Figure 9)** | | |  |
|  | Conditions | CTL | RSV |  |
|  | TeloHAEC | 1.00 ± 0.13 | 2.83 ± 1.18* |  |
|  |  |  |  |  |
|  | **Navβ3 protein (Figure 9)** | | |  |
|  | Conditions | CTL | RSV |  |
|  | TeloHAEC | 1.00 ± 0.18 | 1.67 ± 0.34* |  |
|  |  |  |  |  |

|  |  |
| --- | --- |

**Supplementary Table S2. Densitometry values**

Densitometry analysis of protein expression fold change values normalized to HSC70 (loading control protein) and compared to static condition (Figures 2 and 3), or pCTL (Figure 4), given the arbitrary value of 1.

Densitometry analysis of protein expression fold change values normalized to GAPDH (loading control protein) and compared to control DMSO (CTL) (Figure 9). Results are expressed as mean ± SEM (n=3-7). Non-parametric unpaired Mann-Whitney tests were performed. ns: non-significant, * *p*<0.05, ** *p*<0.01.

Densitometry analysis of protein expression fold change values normalized to mTOR or GAPDH (loading control protein) and compared to static condition with siRNA control (SiCTL) under static condition, given the arbitrary value of 1 (Figure 7). Results are expressed as mean ± SEM (n=4). One-way ANOVA was performed. ns: non-significant, * p<0.05, ** p<0.01, *** p<0.001.
