# Supplementary material for: A Novel Function of NaV Channel β3 Subunit in Endothelial Cell Alignment Through Autophagy Modulation
Source: FASEB J. 2025 May 30;39(11):e70663. doi: 10.1096/fj.202401558RR (PMC12124425; doi:10.1096/fj.202401558RR)
Supplement: Supplementary file 3 — Table S3. [file FSB2-39-e70663-s002.docx]

| **D** | **Name** | **Score** | **Relative  score** | **Sequence  ID** | **Start** | **End** | **Start** | **End** | **Strand** | **Predicted  sequence** | **KLF4  sites** |
| --- | --- | --- | --- | --- | --- | --- | --- | --- | --- | --- | --- |
| [MA0039.4](https://jaspar.elixir.no/matrix/MA0039.4) | MA0039.4.KLF4 | 11.70 | 0.9103 | FP016985 | 1269 | 1280 | -731 | -720 | - | GCCCCCGCCCCA | S1 |
| [MA0039.5](https://jaspar.elixir.no/matrix/MA0039.5) | MA0039.5.KLF4 | 11.76 | 0.9389 | FP016985 | 1271 | 1278 | -729 | -722 | - | CCCCGCCC |  |
| [MA0039.4](https://jaspar.elixir.no/matrix/MA0039.4) | MA0039.4.KLF4 | 11.37 | 0.9036 | FP016985 | 1423 | 1434 | -577 | -566 | + | CCCCCCTCCCCT | S2 |
| [MA0039.5](https://jaspar.elixir.no/matrix/MA0039.5) | MA0039.5.KLF4 | 10.84 | 0.9186 | FP016985 | 1425 | 1432 | -575 | -568 | + | CCCCTCCC |  |
| [MA0039.4](https://jaspar.elixir.no/matrix/MA0039.4) | MA0039.4.KLF4 | 11.23 | 0.9008 | FP016985 | 1899 | 1910 | -101 | -90 | - | TGCCCCTCCCAG | S3 |
| [MA0039.5](https://jaspar.elixir.no/matrix/MA0039.5) | MA0039.5.KLF4 | 10.84 | 0.9186 | FP016985 | 1901 | 1908 | -99 | -92 | - | CCCCTCCC |  |
| [MA0039.4](https://jaspar.elixir.no/matrix/MA0039.4) | MA0039.4.KLF4 | 15.48 | 0.9869 | FP016985 | 1920 | 1931 | -80 | -69 | - | CTCCCCACCCCC | S4 |
| [MA0039.5](https://jaspar.elixir.no/matrix/MA0039.5) | MA0039.5.KLF4 | 14.54 | 0.9999 | FP016985 | 1922 | 1929 | -78 | -71 | - | CCCCACCC |  |
| [MA0039.3](https://jaspar.elixir.no/matrix/MA0039.3) | MA0039.3.KLF4 | 11.55 | 0.9147 | FP016985 | 2105 | 2115 | 105 | 115 | + | CTACACCCACC | S5 |
| [MA0039.5](https://jaspar.elixir.no/matrix/MA0039.5) | MA0039.5.KLF4 | 10.25 | 0.9057 | FP016985 | 2109 | 2116 | 109 | 116 | + | ACCCACCC |  |
| [MA0039.4](https://jaspar.elixir.no/matrix/MA0039.4) | MA0039.4.KLF4 | 12.53 | 0.9272 | FP016985 | 2494 | 2505 | 494 | 505 | - | CTCCCCGCCCAC | S6 |
| [MA0039.5](https://jaspar.elixir.no/matrix/MA0039.5) | MA0039.5.KLF4 | 11.76 | 0.9389 | FP016985 | 2496 | 2503 | 496 | 503 | - | CCCCGCCC |  |
| [MA0039.4](https://jaspar.elixir.no/matrix/MA0039.4) | MA0039.4.KLF4 | 13.48 | 0.9465 | FP016985 | 2606 | 2617 | 606 | 617 | + | GCCCCCACCCGG | S7 |
| [MA0039.5](https://jaspar.elixir.no/matrix/MA0039.5) | MA0039.5.KLF4 | 14.54 | 0.9999 | FP016985 | 2608 | 2615 | 608 | 615 | + | CCCCACCC |  |

**Supplementary Table S3. KLF4 Binding Sites on *SCN3B* promoter and gene**
