# Supplementary material for: A Novel Function of NaV Channel β3 Subunit in Endothelial Cell Alignment Through Autophagy Modulation
Source: FASEB J. 2025 May 30;39(11):e70663. doi: 10.1096/fj.202401558RR (PMC12124425; doi:10.1096/fj.202401558RR)
Supplement: Supplementary file 4 — Table S4. [file FSB2-39-e70663-s001.docx]

| **510  interactants** | **NavB3  HUVEC** | **number  Peptides (95%)** | **510  interactants** | **NavB3  HUVEC** | **number  Peptides (95%)** |
| --- | --- | --- | --- | --- | --- |
| 1 | GCN1L1 | 87 | 48 | BCAP31 | 9 |
| 2 | TRRAP | 44 | 49 | CDK11B | 9 |
| 3 | MAP4K4 | 41 | 50 | GBF1 | 9 |
| 4 | TUBB3 | 36 | 51 | LTN1 | 9 |
| 5 | TJP1 | 31 | 52 | MAN1B1 | 9 |
| 6 | KIAA0368 | 30 | 53 | MDN1 | 9 |
| 7 | CSE1L | 26 | 54 | MTCH1 | 9 |
| 8 | ACTC1 | 25 | 55 | PDS5B | 9 |
| 9 | CKAP5 | 25 | 56 | TNPO3 | 9 |
| 10 | PDS5A | 24 | 57 | XPO5 | 9 |
| 11 | RRP12 | 24 | 58 | HSPH1 | 8 |
| 12 | TEX10 | 24 | 59 | PSME2 | 8 |
| 13 | DENND4C | 23 | 60 | SEC22B | 8 |
| 14 | **MTOR** | **21** | 61 | SYMPK | 8 |
| 15 | UTP20 | 21 | 62 | THADA | 8 |
| 16 | CKAP4 | 19 | 63 | THEM6 | 8 |
| 17 | APMAP | 15 | 64 | ABCB8 | 7 |
| 18 | CPT1A | 15 | 65 | BTAF1 | 7 |
| 19 | CSNK1D | 15 | 66 | CUL4A | 7 |
| 20 | OPA1 | 15 | 67 | EPHA2 | 7 |
| 21 | **SCN3B** | **15** | 68 | FANCI | 7 |
| 22 | MYBBP1A | 14 | 69 | GANAB | 7 |
| 23 | NUP188 | 14 | 70 | HEATR2 | 7 |
| 24 | KRT6A | 13 | 71 | HLA-B | 7 |
| 25 | MON2 | 13 | 72 | LEMD2 | 7 |
| 26 | NF1 | 13 | 73 | LMAN2 | 7 |
| 27 | NCAPD2 | 12 | 74 | MED23 | 7 |
| 28 | ABCC4 | 11 | 75 | MET | 7 |
| 29 | ATE1 | 11 | 76 | MLLT4 | 7 |
| 30 | CANX | 11 | 77 | NOC3L | 7 |
| 31 | GPR126 | 11 | 78 | PEX11B | 7 |
| 32 | GPR126 | 11 | 79 | TAF9 | 7 |
| 33 | HIP1R | 11 | 80 | UBE3C | 7 |
| 34 | INTS7 | 11 | 81 | BYSL | 6 |
| 35 | KRT16 | 11 | 82 | BZW2 | 6 |
| 36 | PPP2R5D | 11 | 83 | DHRS7 | 6 |
| 37 | RBM33 | 11 | 84 | DPY19L1 | 6 |
| 38 | SDAD1 | 11 | 85 | EMC1 | 6 |
| 39 | TMEM43 | 11 | 86 | GOLM1 | 6 |
| 40 | TMTC3 | 11 | 87 | HSD17B12 | 6 |
| 41 | XPO7 | 11 | 88 | KNTC1 | 6 |
| 42 | ABCB10 | 10 | 89 | PNPLA6 | 6 |
| 43 | ATP2B4 | 10 | 90 | PPP2R1B | 6 |
| 44 | NOMO3 | 10 | 91 | PSME1 | 6 |
| 45 | TELO2 | 10 | 92 | RAPGEF1 | 6 |
| 46 | TUBGCP3 | 10 | 93 | SLC25A17 | 6 |
| 47 | XPO6 | 10 | 94 | SLC3A2 | 6 |
| **510  interactants** | **NavB3  HUVEC** | **number  Peptides (95%)** | **510  interactants** | **NavB3  HUVEC** | **number  Peptides (95%)** |
| 95 | SNX8 | 6 | 142 | GALNT1 | 4 |
| 96 | TBCD | 6 | 143 | GLG1 | 4 |
| 97 | TMED9 | 6 | 144 | GPR89A | 4 |
| 98 | TMEM201 | 6 | 145 | HBA1 | 4 |
| 99 | TMEM48 | 6 | 146 | HIST1H1C | 4 |
| 100 | TNFAIP3 | 6 | 147 | HLA-A | 4 |
| 101 | TUBGCP4 | 6 | 148 | IPO4 | 4 |
| 102 | USP24 | 6 | 149 | IQGAP1 | 4 |
| 103 | WFS1 | 6 | 150 | JUP | 4 |
| 104 | ADCK5 | 5 | 151 | NEK6 | 4 |
| 105 | CALM2 | 5 | 152 | PEX16 | 4 |
| 106 | CLCN7 | 5 | 153 | PPP3CB | 4 |
| 107 | CLPTM1 | 5 | 154 | PRAF2 | 4 |
| 108 | DNAJC7 | 5 | 155 | RARS2 | 4 |
| 109 | ERBB2 | 5 | 156 | RBBP7 | 4 |
| 110 | FAM115A | 5 | 157 | SEL1L | 4 |
| 111 | GALNT2 | 5 | 158 | SLC25A24 | 4 |
| 112 | HTRA1 | 5 | 159 | SLC27A4 | 4 |
| 113 | IPO11 | 5 | 160 | STX12 | 4 |
| 114 | NOC2L | 5 | 161 | TGFBR1 | 4 |
| 115 | RANGAP1 | 5 | 162 | TM9SF3 | 4 |
| 116 | SLC35B2 | 5 | 163 | TMEM181 | 4 |
| 117 | SLC35E1 | 5 | 164 | TMEM55B | 4 |
| 118 | SLC38A1 | 5 | 165 | UBC | 4 |
| 119 | TMED4 | 5 | 166 | UQCRH | 4 |
| 120 | TMED7 | 5 | 167 | VTI1B | 4 |
| 121 | TMEM194A | 5 | 168 | WLS | 4 |
| 122 | TMEM214 | 5 | 169 | ZMPSTE24 | 4 |
| 123 | XXYLT1 | 5 | 170 | AAAS | 3 |
| 124 | ABHD16A | 4 | 171 | ABCD1 | 3 |
| 125 | AGPAT2 | 4 | 172 | ADAM10 | 3 |
| 126 | AGPAT5 | 4 | 173 | ATP1B1 | 3 |
| 127 | AGPAT6 | 4 | 174 | CALR | 3 |
| 128 | ALG1 | 4 | 175 | CD97 | 3 |
| 129 | ALG10 | 4 | 176 | CLPTM1L | 3 |
| 130 | AP1G1 | 4 | 177 | CORO7 | 3 |
| 131 | ATP2C1 | 4 | 178 | CSTF2 | 3 |
| 132 | BCAP29 | 4 | 179 | CTBP1 | 3 |
| 133 | CENPN | 4 | 180 | CUL1 | 3 |
| 134 | CNOT1 | 4 | 181 | EARS2 | 3 |
| 135 | DHCR24 | 4 | 182 | EI24 | 3 |
| 136 | DNAJC10 | 4 | 183 | ENDOD1 | 3 |
| 137 | DOCK5 | 4 | 184 | ERGIC1 | 3 |
| 138 | ERLEC1 | 4 | 185 | ERMP1 | 3 |
| 139 | FAM3C | 4 | 186 | EXOC5 | 3 |
| 140 | FKBP10 | 4 | 187 | EXT2 | 3 |
| 141 | FNIP1 | 4 | 188 | EXTL2 | 3 |
| **510  interactants** | **NavB3  HUVEC** | **number  Peptides (95%)** | **510  interactants** | **NavB3  HUVEC** | **number  Peptides (95%)** |
| 189 | FAM57A | 3 | 236 | TMEM115 | 3 |
| 190 | FANCD2 | 3 | 237 | TMEM120A | 3 |
| 191 | FAR2 | 3 | 238 | TMEM126A | 3 |
| 192 | GGCX | 3 | 239 | TOR1AIP1 | 3 |
| 193 | GIMAP1 | 3 | 240 | TRABD | 3 |
| 194 | GJA1 | 3 | 241 | TRAM2 | 3 |
| 195 | GNAS | 3 | 242 | TRAPPC5 | 3 |
| 196 | GOLIM4 | 3 | 243 | TTI1 | 3 |
| 197 | IGF2R | 3 | 244 | TXN | 3 |
| 198 | IGFBP3 | 3 | 245 | UNC84A | 3 |
| 199 | INTS6 | 3 | 246 | URB2 | 3 |
| 200 | ITGA3 | 3 | 247 | VARS | 3 |
| 201 | KIDINS220 | 3 | 248 | VPS51 | 3 |
| 202 | LMF2 | 3 | 249 | ZW10 | 3 |
| 203 | LPCAT4 | 3 | 250 | ACAP2 | 2 |
| 204 | MED24 | 3 | 251 | APOOL | 2 |
| 205 | MGAT2 | 3 | 252 | APP | 2 |
| 206 | MLEC | 3 | 253 | ARFGEF2 | 2 |
| 207 | MT-CO2 | 3 | 254 | ARL8B | 2 |
| 208 | MYO19 | 3 | 255 | AURKB | 2 |
| 209 | NGLY1 | 3 | 256 | BET1 | 2 |
| 210 | NOC4L | 3 | 257 | CANT1 | 2 |
| 211 | OAT | 3 | 258 | CENPL | 2 |
| 212 | P4HA2 | 3 | 259 | CHTF18 | 2 |
| 213 | PIGG | 3 | 260 | CLASP2 | 2 |
| 214 | PPP3CC | 3 | 261 | CLN8 | 2 |
| 215 | PSEN1 | 3 | 262 | COG4 | 2 |
| 216 | PUM1 | 3 | 263 | DCAF10 | 2 |
| 217 | RAB5A | 3 | 264 | DNAJB2 | 2 |
| 218 | RABL3 | 3 | 265 | DNAJC19 | 2 |
| 219 | RANBP6 | 3 | 266 | DOCK1 | 2 |
| 220 | RBFOX1 | 3 | 267 | EDEM3 | 2 |
| 221 | RETSAT | 3 | 268 | EFNB1 | 2 |
| 222 | RMDN3 | 3 | 269 | EIF2B1 | 2 |
| 223 | RPS26P11 | 3 | 270 | EIF2B3 | 2 |
| 224 | SGPL1 | 3 | 271 | ERAP1 | 2 |
| 225 | SHMT2 | 3 | 272 | EXOC1 | 2 |
| 226 | SIRPA | 3 | 273 | EXOC2 | 2 |
| 227 | SMPD4 | 3 | 274 | FAM105A | 2 |
| 228 | STK24 | 3 | 275 | GEMIN4 | 2 |
| 229 | SUN2 | 3 | 276 | GLCE | 2 |
| 230 | SYNJ2BP | 3 | 277 | GMPPA | 2 |
| 231 | TAF6 | 3 | 278 | H3F3B | 2 |
| 232 | TAP2 | 3 | 279 | HAX1 | 2 |
| 233 | TM9SF2 | 3 | 280 | HS2ST1 | 2 |
| 234 | TMED2 | 3 | 281 | IPO8 | 2 |
| 235 | TMEM106B | 3 | 282 | ITGB1 | 2 |
| **510  interactants** | **NavB3  HUVEC** | **number  Peptides (95%)** | **510  interactants** | **NavB3  HUVEC** | **number  Peptides (95%)** |
| 283 | KCT2 | 2 | 330 | SLC25A51 | 2 |
| 284 | KIAA2013 | 2 | 331 | SLC30A7 | 2 |
| 285 | LETMD1 | 2 | 332 | SLC39A14 | 2 |
| 286 | LRRC40 | 2 | 333 | SLC5A6 | 2 |
| 287 | LRRC41 | 2 | 334 | SMEK1 | 2 |
| 288 | MAN1A2 | 2 | 335 | SOAT1 | 2 |
| 289 | MANEA | 2 | 336 | SRPR | 2 |
| 290 | MED16 | 2 | 337 | SRSF4 | 2 |
| 291 | MFF | 2 | 338 | ST3GAL1 | 2 |
| 292 | MLF1 | 2 | 339 | STRIP1 | 2 |
| 293 | MLST8 | 2 | 340 | TAP1 | 2 |
| 294 | MMP14 | 2 | 341 | TARDBP | 2 |
| 295 | MPRIP | 2 | 342 | TM9SF4 | 2 |
| 296 | MRPL10 | 2 | 343 | TMCC3 | 2 |
| 297 | MUL1 | 2 | 344 | TMCO1 | 2 |
| 298 | NCAPG2 | 2 | 345 | TMEM11 | 2 |
| 299 | NDUFV1 | 2 | 346 | TMEM135 | 2 |
| 300 | ODF2L | 2 | 347 | TMEM41A | 2 |
| 301 | OS9 | 2 | 348 | TOMM70A | 2 |
| 302 | PA2G4 | 2 | 349 | TOR1AIP2 | 2 |
| 303 | PDHA1 | 2 | 350 | TRAM1 | 2 |
| 304 | PELP1 | 2 | 351 | UBAC2 | 2 |
| 305 | PEX10 | 2 | 352 | UNC93B1 | 2 |
| 306 | PEX3 | 2 | 353 | VPS39 | 2 |
| 307 | PIK3C3 | 2 | 354 | ZBTB1 | 2 |
| 308 | PLD3 | 2 | 355 | ZFPL1 | 2 |
| 309 | PLXNB2 | 2 | 356 | ZNF519 | 2 |
| 310 | POLR2J2 | 2 | 357 | ABCB6 | 1 |
| 311 | PPP2R5B | 2 | 358 | ACAA1 | 1 |
| 312 | PPP4R1 | 2 | 359 | ACSL4 | 1 |
| 313 | PSMD13 | 2 | 360 | ADSS | 1 |
| 314 | PTPMT1 | 2 | 361 | AGPS | 1 |
| 315 | QSOX2 | 2 | 362 | ALG3 | 1 |
| 316 | RAB18 | 2 | 363 | AMFR | 1 |
| 317 | RAB20 | 2 | 364 | AP5Z1 | 1 |
| 318 | RAB7L1 | 2 | 365 | API5 | 1 |
| 319 | RAP1B | 2 | 366 | ARFGEF1 | 1 |
| 320 | RINT1 | 2 | 367 | ARHGDIA | 1 |
| 321 | RNF121 | 2 | 368 | ARL6IP5 | 1 |
| 322 | RNF170 | 2 | 369 | ARPC4-TTLL3 | 1 |
| 323 | RRBP1 | 2 | 370 | ARVCF | 1 |
| 324 | RTN3 | 2 | 371 | ATXN10 | 1 |
| 325 | SAMM50 | 2 | 372 | AUP1 | 1 |
| 326 | SCAMP1 | 2 | 373 | B2M | 1 |
| 327 | SCAMP2 | 2 | 374 | BAG6 | 1 |
| 328 | SCD | 2 | 375 | BRAT1 | 1 |
| 329 | SEC22A | 2 | 376 | BRI3BP | 1 |
| **510  interactants** | **NavB3  HUVEC** | **number  Peptides (95%)** | **510  interactants** | **NavB3  HUVEC** | **number  Peptides (95%)** |
| 377 | C14orf1 | 1 | 468 | RHBDD1 | 1 |
| 378 | CALD1 | 1 | 469 | RMDN2 | 1 |
| 379 | CASC4 | 1 | 470 | RNF216 | 1 |
| 380 | CCDC82 | 1 | 471 | ROMO1 | 1 |
| 381 | CCRN4L | 1 | 472 | RPTOR | 1 |
| 382 | CD151 | 1 | 473 | RTN4 | 1 |
| 383 | CDC27 | 1 | 474 | S1PR1 | 1 |
| 384 | CDKAL1 | 1 | 475 | SCD5 | 1 |
| 385 | CHERP | 1 | 476 | SELK | 1 |
| 386 | CNIH4 | 1 | 477 | SEMA3C | 1 |
| 387 | COA1 | 1 | 478 | SLC1A5 | 1 |
| 388 | COQ5 | 1 | 479 | SLC25A38 | 1 |
| 389 | COX6C | 1 | 480 | SLC35A3 | 1 |
| 390 | CRIPT | 1 | 481 | SND1 | 1 |
| 391 | CRTAP | 1 | 482 | SPPL2B | 1 |
| 392 | CYB561D2 | 1 | 483 | ST6GALNAC4 | 1 |
| 393 | DAD1 | 1 | 484 | STK4 | 1 |
| 394 | DCTN5 | 1 | 485 | STX5 | 1 |
| 395 | DDX19B | 1 | 486 | SUPT7L | 1 |
| 396 | DEGS1 | 1 | 487 | TANGO6 | 1 |
| 397 | DHFR | 1 | 488 | TAZ | 1 |
| 398 | DLST | 1 | 489 | TFB1M | 1 |
| 399 | DNAJB9 | 1 | 490 | THOC2 | 1 |
| 400 | ECH1 | 1 | 491 | TM7SF2 | 1 |
| 401 | EDC4 | 1 | 492 | TMED5 | 1 |
| 402 | EEF1B2 | 1 | 493 | TMEM101 | 1 |
| 403 | EMC3 | 1 | 494 | TMEM120B | 1 |
| 404 | ENTPD4 | 1 | 495 | TMEM245 | 1 |
| 405 | EXOC7 | 1 | 496 | TMEM9 | 1 |
| 406 | FADS3 | 1 | 497 | TMPPE | 1 |
| 407 | FAM179B | 1 | 498 | TNKS1BP1 | 1 |
| 408 | FAM213A | 1 | 499 | TOR1B | 1 |
| 409 | FAM32A | 1 | 500 | TRIM24 | 1 |
| 410 | FAM69B | 1 | 501 | TRIM4 | 1 |
| 411 | FAM96B | 1 | 502 | TTI2 | 1 |
| 412 | FKBP11 | 1 | 503 | TTK | 1 |
| 413 | FLVCR1 | 1 | 504 | TUSC3 | 1 |
| 414 | G3BP2 | 1 | 505 | VPS26A | 1 |
| 415 | GALNT6 | 1 | 506 | WRB | 1 |
| 416 | GIPC1 | 1 | 507 | WWC1 | 1 |
| 417 | GLB1L2 | 1 | 508 | YIPF6 | 1 |
| 418 | GLT8D1 | 1 | 509 | ZDHHC13 | 1 |
| 419 | GPC1 | 1 | 510 | ZNF556 | 1 |
| 420 | GPR107 | 1 |  |  |  |
| 421 | GPR180 | 1 |  |  |  |
| 422 | GPRC5B | 1 |  |  |  |
| 423 | GTF2I | 1 |  |  |  |
| **510  interactants** | **NavB3  HUVEC** | **number  Peptides (95%)** |  |  |  |
| 424 | HDLBP | 1 |  |  |  |
| 425 | HEATR5B | 1 |  |  |  |
| 426 | HERC2 | 1 |  |  |  |
| 427 | HSD17B13 | 1 |  |  |  |
| 428 | HTT | 1 |  |  |  |
| 429 | INTS4 | 1 |  |  |  |
| 430 | ITGA5 | 1 |  |  |  |
| 431 | KDELC1 | 1 |  |  |  |
| 432 | KDM1A | 1 |  |  |  |
| 433 | KIAA0355 | 1 |  |  |  |
| 434 | KIAA1524 | 1 |  |  |  |
| 435 | KRTCAP2 | 1 |  |  |  |
| 436 | LCLAT1 | 1 |  |  |  |
| 437 | LPCAT1 | 1 |  |  |  |
| 438 | LPL | 1 |  |  |  |
| 439 | LSM3 | 1 |  |  |  |
| 440 | MBLAC2 | 1 |  |  |  |
| 441 | MFSD10 | 1 |  |  |  |
| 442 | MGST2 | 1 |  |  |  |
| 443 | NAPRT1 | 1 |  |  |  |
| 444 | NAT14 | 1 |  |  |  |
| 445 | NBAS | 1 |  |  |  |
| 446 | NCDN | 1 |  |  |  |
| 447 | NDUFS8 | 1 |  |  |  |
| 448 | NFS1 | 1 |  |  |  |
| 449 | NIPA1 | 1 |  |  |  |
| 450 | NME3 | 1 |  |  |  |
| 451 | NT5C3 | 1 |  |  |  |
| 452 | OXA1L | 1 |  |  |  |
| 453 | PAPD5 | 1 |  |  |  |
| 454 | PGRMC2 | 1 |  |  |  |
| 455 | PHKA1 | 1 |  |  |  |
| 456 | PIGW | 1 |  |  |  |
| 457 | POFUT1 | 1 |  |  |  |
| 458 | PPP2R4 | 1 |  |  |  |
| 459 | PTGES2 | 1 |  |  |  |
| 460 | PTPRF | 1 |  |  |  |
| 461 | RAB7A | 1 |  |  |  |
| 462 | RAC1 | 1 |  |  |  |
| 463 | RAC2 | 1 |  |  |  |
| 464 | RAD51 | 1 |  |  |  |
| 465 | RALGAPA1 | 1 |  |  |  |
| 466 | RBM12 | 1 |  |  |  |
| 467 | RBM14/RBM4 | 1 |  |  |  |

| **289 interactants** | **NavB3  TeloHAEC** | **number  Peptides (95%)** | **289 interactants** | **NavB3  TeloHAEC** | **number  Peptides (95%)** |
| --- | --- | --- | --- | --- | --- |
| 1 | PRKDC | 74 | 48 | CSE1L | 5 |
| 2 | CEP170 | 65 | 49 | TNPO1 | 5 |
| 3 | GCN1L1 | 64 | 50 | ACSL1 | 5 |
| 4 | DENND4C | 50 | 51 | PHB | 5 |
| 5 | MAP4K4 | 48 | 52 | SMC1A | 5 |
| 6 | MAP4K4 | 47 | 53 | ABCD3 | 5 |
| 7 | NHSL1 | 38 | 54 | PLEKHO1 | 5 |
| 8 | MTHFD1 | 27 | 55 | WLS | 5 |
| 9 | KRT6A | 18 | 56 | TAP2 | 5 |
| 10 | KRT6B | 16 | 57 | TMCC3 | 5 |
| 11 | CSNK1D | 15 | 58 | SEC22B | 4 |
| 12 | CPT1A | 14 | 59 | SUN2 | 4 |
| 13 | KIAA0368 | 13 | 60 | TPM3 | 4 |
| 14 | **MTOR** | **12** | 61 | TMPO | 4 |
| 15 | CCT7 | 12 | 62 | RPN2 | 4 |
| 16 | FAM21C | 11 | 63 | RPGRIP1L | 4 |
| 17 | ATE1 | 11 | 64 | DDOST | 4 |
| 18 | **SCN3B** | **10** | 65 | RPL36AL | 4 |
| 19 | ANKHD1 | 10 | 66 | PPP3CA | 4 |
| 20 | RPL26L1 | 10 | 67 | PLXNA2 | 4 |
| 21 | CCT3 | 10 | 68 | SURF4 | 4 |
| 22 | LTN1 | 9 | 69 | APMAP | 4 |
| 23 | CKAP4 | 9 | 70 | CANX | 4 |
| 24 | FGD6 | 9 | 71 | NUP160 | 4 |
| 25 | HLA-B | 9 | 72 | TMEM165 | 4 |
| 26 | MON2 | 8 | 73 | DDX60L | 4 |
| 27 | CRYBG3 | 8 | 74 | PTPN21 | 4 |
| 28 | ANKRD50 | 8 | 75 | KRT18 | 4 |
| 29 | THADA | 8 | 76 | DAB2IP | 4 |
| 30 | RPN1 | 7 | 77 | KIF1C | 4 |
| 31 | NUP93 | 7 | 78 | FAM188B | 4 |
| 32 | IPO7 | 7 | 79 | ZMYM1 | 4 |
| 33 | COPG2 | 7 | 80 | MYL12A | 4 |
| 34 | HSP90AA1 | 7 | 81 | DNM1L | 4 |
| 35 | ACSL3 | 7 | 82 | ALG1 | 4 |
| 36 | FASN | 7 | 83 | STX12 | 4 |
| 37 | TAP1 | 7 | 84 | RTN4 | 4 |
| 38 | RAPGEF6 | 6 | 85 | TRIM24 | 4 |
| 39 | SYMPK | 6 | 86 | MYBBP1A | 3 |
| 40 | XPO1 | 6 | 87 | ESYT2 | 3 |
| 41 | PPP1CA | 6 | 88 | COG5 | 3 |
| 42 | CCT4 | 6 | 89 | PHACTR2 | 3 |
| 43 | CDK9 | 6 | 90 | TMED10 | 3 |
| 44 | AGO1 | 6 | 91 | DYRK1A | 3 |
| 45 | BRCA2 | 6 | 92 | MBOAT7 | 3 |
| 46 | SIRPA | 6 | 93 | PDZD11 | 3 |
| 47 | S1PR1 | 6 | 94 | CDC37 | 3 |
| **289 interactants** | **NavB3  TeloHAEC** | **number  Peptides (95%)** | **289 interactants** | **NavB3  TeloHAEC** | **number  Peptides (95%)** |
| 95 | FNDC3A | 3 | 142 | EXOSC2 | 2 |
| 96 | SDF4 | 3 | 143 | GPX8 | 2 |
| 97 | RPS29 | 3 | 144 | POLR3E | 2 |
| 98 | PXDN | 3 | 145 | TMEM214 | 2 |
| 99 | CDIPT | 3 | 146 | TRIM3 | 2 |
| 100 | HMGCL | 3 | 147 | WWC3 | 2 |
| 101 | CSPP1 | 3 | 148 | ACVRL1 | 2 |
| 102 | USP13 | 3 | 149 | FN1 | 2 |
| 103 | SUB1 | 3 | 150 | LSM7 | 2 |
| 104 | IKBKB | 3 | 151 | CHD9 | 2 |
| 105 | PKN2 | 3 | 152 | MTHFD2 | 2 |
| 106 | TMEM33 | 3 | 153 | PSMD4 | 2 |
| 107 | C9orf72 | 3 | 154 | TNFRSF10B | 2 |
| 108 | RFC5 | 3 | 155 | DDX58 | 2 |
| 109 | HNRNPA2B1 | 3 | 156 | CAND1 | 2 |
| 110 | AP3S2 | 3 | 157 | DCAF7 | 2 |
| 111 | PFKL | 3 | 158 | CD70 | 2 |
| 112 | TMED9 | 3 | 159 | SLC39A10 | 2 |
| 113 | RETSAT | 3 | 160 | HMGCR | 2 |
| 114 | LPCAT4 | 3 | 161 | NPM1 | 2 |
| 115 | ATXN10 | 3 | 162 | ACO1 | 2 |
| 116 | TMED2 | 2 | 163 | ZMYM2 | 2 |
| 117 | HEATR2 | 2 | 164 | CNOT1 | 2 |
| 118 | TMEM43 | 2 | 165 | DYNC1I2 | 2 |
| 119 | UBE3C | 2 | 166 | IL13RA2 | 2 |
| 120 | SPTLC1 | 2 | 167 | ANKZF1 | 2 |
| 121 | EXOC4 | 2 | 168 | PARP9 | 2 |
| 122 | PHB2 | 2 | 169 | RAB32 | 2 |
| 123 | IDH2 | 2 | 170 | GCFC2 | 2 |
| 124 | ZNF598 | 2 | 171 | ARHGAP24 | 2 |
| 125 | ATP1A1 | 2 | 172 | LMF2 | 2 |
| 126 | UGDH | 2 | 173 | PSMD6 | 2 |
| 127 | NACA | 2 | 174 | FADS2 | 2 |
| 128 | DHRS7 | 2 | 175 | WDR53 | 2 |
| 129 | PHKG2 | 2 | 176 | HM13 | 2 |
| 130 | OSBPL11 | 2 | 177 | IARS | 2 |
| 131 | CYP2S1 | 2 | 178 | LRCH1 | 2 |
| 132 | LGALS9 | 2 | 179 | SRPK2 | 2 |
| 133 | TCEB1 | 2 | 180 | TACC3 | 2 |
| 134 | USP16 | 2 | 181 | MYO10 | 2 |
| 135 | RER1 | 2 | 182 | GTSE1 | 2 |
| 136 | FAR1 | 2 | 183 | FAR2 | 2 |
| 137 | C1orf109 | 2 | 184 | ZBTB1 | 2 |
| 138 | TKT | 2 | 185 | MLST8 | 2 |
| 139 | RARS | 2 | 186 | SLC30A7 | 2 |
| 140 | TUBGCP4 | 2 | 187 | EXOC1 | 2 |
| 141 | SKA3 | 2 | 188 | B2M | 2 |
| **289 interactants** | **NavB3  TeloHAEC** | **number  Peptides (95%)** | **289 interactants** | **NavB3  TeloHAEC** | **number  Peptides (95%)** |
| 189 | SCD5 | 2 | 236 | PER1 | 1 |
| 190 | ARFGEF1 | 2 | 237 | DSN1 | 1 |
| 191 | FAM179B | 2 | 238 | MAPKBP1 | 1 |
| 192 | BCAP31 | 1 | 239 | ANGPTL4 | 1 |
| 193 | RARS2 | 1 | 240 | PELO | 1 |
| 194 | NF1 | 1 | 241 | SFXN3 | 1 |
| 195 | ATP2C1 | 1 | 242 | DNAJC1 | 1 |
| 196 | XXYLT1 | 1 | 243 | ME2 | 1 |
| 197 | ARFGEF2 | 1 | 244 | ARF6 | 1 |
| 198 | PPP2R1B | 1 | 245 | TOR4A | 1 |
| 199 | PSME1 | 1 | 246 | PPP2R5B | 1 |
| 200 | CDK11B | 1 | 247 | DNAJB4 | 1 |
| 201 | XPO5 | 1 | 248 | RCN3 | 1 |
| 202 | BCAP29 | 1 | 249 | CTTN | 1 |
| 203 | ENDOD1 | 1 | 250 | SIK2 | 1 |
| 204 | TMED7 | 1 | 251 | CLASP2 | 1 |
| 205 | TMEM126A | 1 | 252 | TP53BP1 | 1 |
| 206 | RBM4 | 1 | 253 | NRP2 | 1 |
| 207 | POLR2H | 1 | 254 | MECR | 1 |
| 208 | RAI14 | 1 | 255 | ANKRD52 | 1 |
| 209 | LASP1 | 1 | 256 | EXOSC7 | 1 |
| 210 | TTC27 | 1 | 257 | CDS2 | 1 |
| 211 | CALM3 | 1 | 258 | ELMO2 | 1 |
| 212 | SPARC | 1 | 259 | MMRN1 | 1 |
| 213 | XPOT | 1 | 260 | FAM207A | 1 |
| 214 | FBN2 | 1 | 261 | USP18 | 1 |
| 215 | PRICKLE2 | 1 | 262 | ABHD10 | 1 |
| 216 | PNO1 | 1 | 263 | IPO9 | 1 |
| 217 | CDC42EP1 | 1 | 264 | NSL1 | 1 |
| 218 | HNRNPC | 1 | 265 | FAM122B | 1 |
| 219 | SDF2L1 | 1 | 266 | PMF1 | 1 |
| 220 | SH3RF3 | 1 | 267 | FLG2 | 1 |
| 221 | PLOD2 | 1 | 268 | STK3 | 1 |
| 222 | GNAI2 | 1 | 269 | NTPCR | 1 |
| 223 | SH2D3C | 1 | 270 | ACOT7 | 1 |
| 224 | CYTH3 | 1 | 271 | ICAM2 | 1 |
| 225 | LDHB | 1 | 272 | MAP2K3 | 1 |
| 226 | IQGAP2 | 1 | 273 | MRPL22 | 1 |
| 227 | PSMD10 | 1 | 274 | RAB11A | 1 |
| 228 | SMARCAD1 | 1 | 275 | ERGIC2 | 1 |
| 229 | ABCD1 | 1 | 276 | FOXP4 | 1 |
| 230 | GIMAP1 | 1 | 277 | ZWINT | 1 |
| 231 | ZFYVE26 | 1 | 278 | NKIRAS1 | 1 |
| 232 | DERL1 | 1 | 279 | ELOVL1 | 1 |
| 233 | EI24 | 1 | 280 | TMEM109 | 1 |
| 234 | TMEM189 | 1 | 281 | CALCRL | 1 |
| 235 | CCT6A | 1 | 282 | SMTN | 1 |
| **289 interactants** | **NavB3  TeloHAEC** | **number  Peptides (95%)** |  |  |  |
| 283 | MGST2 | 1 |  |  |  |
| 284 | KRTCAP2 | 1 |  |  |  |
| 285 | S100A6 | 1 |  |  |  |
| 286 | ITGA5 | 1 |  |  |  |
| 287 | OOEP | 1 |  |  |  |
| 288 | MARS | 1 |  |  |  |
| 289 | SREBF1 | 1 |  |  |  |

| **76 interactants** | **NavB3  HUVEC** | **number  Peptides (95%)** | **NavB3  TeloHAEC** | **number  Peptides (95%)** |
| --- | --- | --- | --- | --- |
| 1 | GCN1L1 | 87 | GCN1L1 | 64 |
| 2 | MAP4K4 | 41 | MAP4K4 | 48 |
| 3 | KIAA0368 | 30 | KIAA0368 | 13 |
| 4 | CSE1L | 26 | CSE1L | 5 |
| 5 | DENND4C | 23 | DENND4C | 50 |
| 6 | **MTOR** | **21** | **MTOR** | **12** |
| 7 | CKAP4 | 19 | CKAP4 | 9 |
| 8 | APMAP | 15 | APMAP | 4 |
| 9 | CPT1A | 15 | CPT1A | 14 |
| 10 | CSNK1D | 15 | CSNK1D | 15 |
| 11 | **SCN3B** | **15** | **SCN3B** | **10** |
| 12 | MYBBP1A | 14 | MYBBP1A | 3 |
| 13 | KRT6A | 13 | KRT6A | 18 |
| 14 | MON2 | 13 | MON2 | 8 |
| 15 | NF1 | 13 | NF1 | 1 |
| 16 | ATE1 | 11 | ATE1 | 11 |
| 17 | CANX | 11 | CANX | 4 |
| 18 | TMEM43 | 11 | TMEM43 | 2 |
| 19 | BCAP31 | 9 | BCAP31 | 1 |
| 20 | CDK11B | 9 | CDK11B | 1 |
| 21 | LTN1 | 9 | LTN1 | 9 |
| 22 | XPO5 | 9 | XPO5 | 1 |
| 23 | SEC22B | 8 | SEC22B | 4 |
| 24 | SYMPK | 8 | SYMPK | 6 |
| 25 | THADA | 8 | THADA | 8 |
| 26 | HEATR2 | 7 | HEATR2 | 2 |
| 27 | HLA-B | 7 | HLA-B | 9 |
| 28 | UBE3C | 7 | UBE3C | 2 |
| 29 | DHRS7 | 6 | DHRS7 | 2 |
| 30 | PPP2R1B | 6 | PPP2R1B | 1 |
| 31 | PSME1 | 6 | PSME1 | 1 |
| 32 | TMED9 | 6 | TMED9 | 3 |
| 33 | TUBGCP4 | 6 | TUBGCP4 | 2 |
| 34 | TMED7 | 5 | TMED7 | 1 |
| 35 | TMEM214 | 5 | TMEM214 | 2 |
| 36 | XXYLT1 | 5 | XXYLT1 | 1 |
| 37 | ALG1 | 4 | ALG1 | 4 |
| 38 | ATP2C1 | 4 | ATP2C1 | 1 |
| 39 | BCAP29 | 4 | BCAP29 | 1 |
| 40 | CNOT1 | 4 | CNOT1 | 2 |
| 41 | RARS2 | 4 | RARS2 | 1 |
| 42 | STX12 | 4 | STX12 | 4 |
| 43 | WLS | 4 | WLS | 5 |
| 44 | ABCD1 | 3 | ABCD1 | 1 |
| 45 | EI24 | 3 | EI24 | 1 |
| 46 | ENDOD1 | 3 | ENDOD1 | 1 |
| 47 | FAR2 | 3 | FAR2 | 2 |
| **76 interactants** | **NavB3  HUVEC** | **number  Peptides (95%)** | **NavB3  TeloHAEC** | **number  Peptides (95%)** |
| 48 | GIMAP1 | 3 | GIMAP1 | 1 |
| 49 | LMF2 | 3 | LMF2 | 2 |
| 50 | LPCAT4 | 3 | LPCAT4 | 3 |
| 51 | RETSAT | 3 | RETSAT | 3 |
| 52 | SIRPA | 3 | SIRPA | 6 |
| 53 | SUN2 | 3 | SUN2 | 4 |
| 54 | TAP2 | 3 | TAP2 | 5 |
| 55 | TMED2 | 3 | TMED2 | 2 |
| 56 | TMEM126A | 3 | TMEM126A | 1 |
| 57 | ARFGEF2 | 2 | ARFGEF2 | 1 |
| 58 | CLASP2 | 2 | CLASP2 | 1 |
| 59 | EXOC1 | 2 | EXOC1 | 2 |
| 60 | **MLST8** | **2** | **MLST8** | **2** |
| 61 | PPP2R5B | 2 | PPP2R5B | 1 |
| 62 | SLC30A7 | 2 | SLC30A7 | 2 |
| 63 | TAP1 | 2 | TAP1 | 7 |
| 64 | TMCC3 | 2 | TMCC3 | 5 |
| 65 | ZBTB1 | 2 | ZBTB1 | 2 |
| 66 | ARFGEF1 | 1 | ARFGEF1 | 2 |
| 67 | ATXN10 | 1 | ATXN10 | 3 |
| 68 | B2M | 1 | B2M | 2 |
| 69 | FAM179B | 1 | FAM179B | 2 |
| 70 | ITGA5 | 1 | ITGA5 | 1 |
| 71 | KRTCAP2 | 1 | KRTCAP2 | 1 |
| 72 | MGST2 | 1 | MGST2 | 1 |
| 73 | RTN4 | 1 | RTN4 | 4 |
| 74 | S1PR1 | 1 | S1PR1 | 6 |
| 75 | SCD5 | 1 | SCD5 | 2 |
| 76 | TRIM24 | 1 | TRIM24 | 4 |

**Supplementary Table S4. List of the 510 proteins that potentially interact with Na_V_β3 detected in HUVEC, list of the 289 proteins that potentially interact with Na_V_β3 detected in TeloHAEC and list of the 76 proteins in common in HUVEC and TeloHAEC including 11 described in NCBI bank (in red).**
