# Supplementary material for: A Novel Function of NaV Channel β3 Subunit in Endothelial Cell Alignment Through Autophagy Modulation
Source: FASEB J. 2025 May 30;39(11):e70663. doi: 10.1096/fj.202401558RR (PMC12124425; doi:10.1096/fj.202401558RR)
Supplement: Supplementary file 5 — Table S5. [file FSB2-39-e70663-s006.docx]

|  | Experiment 1 | |  | Experiment 2 | |  | | Experiment 3 | | |
| --- | --- | --- | --- | --- | --- | --- | --- | --- | --- | --- |
|  | **LC3B puncta** | |  | **LC3B puncta** | |  | | **LC3B puncta** | | |
| # Field | siCTL | siSCN3B |  | SiCTL | siSCN3B |  | | SiCTL | siSCN3B | |
| 1 | 72 | 45 |  | 220 | 87 |  | | 258 | 175 | |
| 2 | 107 | 57 |  | 200 | 76 |  | | 199 | 114 | |
| 3 | 73 | 69 |  | 157 | 76 |  | | 241 | 148 | |
| 4 | 118 | 57 |  | 193 | 105 |  | | 222 | 187 | |
| 5 | 85 | 91 |  | 213 | 70 |  | | 274 | 163 | |
| 6 | 122 | 78 |  | 231 | 83 |  | | 186 | 157 | |
| 7 | 73 | 89 |  | 221 | 64 |  | | 302 | 129 | |
| 8 | 119 | 47 |  | 204 | 94 |  | | 265 | 143 | |
| 9 | 111 | 54 |  | 140 | 107 |  | | 209 | 139 | |
| 10 | 76 | 64 |  |  | 63 |  | |  |  | |
| mean | 96 | 65 |  | 198 | 83 |  | | 240 | 151 | |
| **ratio** | **1,0** | **0,68** |  | **1,0** | **0,42** |  | | **1,0** | **0,63** | |
|  |  |  |  |  |  |  | |  |  | |
|  | **siCTL** | **siSCN3B** |  |  |  |  | | **siCTL** | **siSCN3B** | |
| **mean of number  of puncta** | **178** | **99** |  |  |  | **mean of ratio** | | **1,00** | **0,58** | |
| **SEM** | **43** | **26** |  |  |  | **SEM** | | **0,00** | **0,08** | |
|  |  |  |  |  |  |  | |  |  | |
|  |  |  |  |  |  |  | |  |  | |
|  | Experiment 1 | |  | Experiment 2 | |  | | Experiment 3 | | |
|  | **Fluorescence intensity** | |  | **Fluorescence intensity** | |  | | **Fluorescence intensity** | | |
| # Field | siCTL | siSCN3B |  | SiCTL | siSCN3B |  | | siCTL | siSCN3B | |
| 1 | 15.38 | 7.62 |  | 148.86 | 81.28 |  | | 98.98 | 32.28 | |
| 2 | 19.10 | 7.10 |  | 173.77 | 86.81 |  | | 105.3 | 86.77 | |
| 3 | 13.07 | 5.51 |  | 151.30 | 77.94 |  | | 104.7 | 88.26 | |
| 4 | 31.60 | 12.14 |  | 143.43 | 161.21 |  | | 76.06 | 58.2 | |
| 5 | 28.41 | 7.23 |  | 182.54 | 72.78 |  | | 81.21 | 25.95 | |
| 6 | 25.70 | 9.96 |  | 234.45 | 162.18 |  | | 119,00 | 57.14 | |
| 7 | 25.01 | 6.02 |  | 140.45 | 163.60 |  | | 97.45 |  | |
| 8 | 23.89 | 10.18 |  | 163.93 | 133.01 |  | |  |  | |
| 9 | 19.34 | 6.27 |  | 164.99 | 104.87 |  | |  |  | |
| 10 | 13.81 | 10.78 |  |  |  |  | |  |  | |
| mean | 21.53 | 8.28 |  | 167.00 | 115.96 |  | | 97.52 | 58.1 | |
| **ratio** | **1,00** | **0.38** |  | **1,00** | **0.69** |  | | **1,00** | **0.59** | |
|  |  |  |  |  |  |  | |  |  | |
|  | **siCTL** | **siSCN3B** |  |  |  |  | | **siCTL** | **siSCN3B** | |
| **mean of fluorescence intensity** | **95.3** | **60.78** |  |  |  | **mean of ratio** | | **1,00** | **0.55** | |
| **SEM** | **42.03** | **31.11** |  |  |  | **SEM** | | **0,00** | **0.09** | |
| **Supplementary Table S5. Number of puncta and fluorescence intensity of LC3B staining from three independent experiments.** | | | | | | |  | | |  |
