# Supplementary material for: A Novel Function of NaV Channel β3 Subunit in Endothelial Cell Alignment Through Autophagy Modulation
Source: FASEB J. 2025 May 30;39(11):e70663. doi: 10.1096/fj.202401558RR (PMC12124425; doi:10.1096/fj.202401558RR)
Supplement: Supplementary file 6 — Table S6. [file FSB2-39-e70663-s003.docx]

| **PCC values** | **Static** | | | **LSS** | | | |  |  |
| --- | --- | --- | --- | --- | --- | --- | --- | --- | --- |
| **Na_V_β3** | **LC3B** | **LAMP1** | **mTOR** | **LC3B** | **LAMP1** | **mTOR** | |  |  |
| Cell 1 |  | 0.438 | 0.387 |  | 0.638 | 0.473 | |  |  |
| Cell 2 |  | 0.402 | 0.326 |  | 0.457 | 0.301 | |  |  |
| Cell 3 |  | 0.512 | 0.368 |  | 0.382 | 0.368 | |  |  |
| Cell 4 |  | 0.393 | 0.272 |  | 0.492 | 0.172 | |  |  |
| Cell 5 |  | 0.436 | 0.38 |  | 0.494 | 0.48 | |  |  |
| **Mean Exp 1** |  | **0.436** | **0.347** |  | **0.492** | **0.359** | |  |  |
|  |  |  |  |  |  |  | |  |  |
| **PCC values** | **Static** | | | **LSS** | | | |  |  |
| **Na_V_β3** | **LC3B** | **LAMP1** | **mTOR** | **LC3B** | **LAMP1** | **mTOR** | |  |  |
| Cell 1 | 0.248 |  |  | 0.322 |  |  | |  |  |
| Cell 2 | 0.124 |  |  | 0.182 |  |  | |  |  |
| Cell 3 | 0.284 |  |  | 0.19 |  |  | |  |  |
| Cell 4 | 0.284 |  |  | 0.266 |  |  | |  |  |
| Cell 5 | 0.344 |  |  | 0.246 |  |  | |  |  |
| Cell 6 | 0.347 |  |  | 0.196 |  |  | |  |  |
| Cell 7 | 0.17 |  |  | 0.167 |  |  | |  |  |
| Cell 8 | 0.26 |  |  | 0.251 |  |  | |  |  |
| Cell 9 | 0.34 |  |  | 0.26 |  |  | |  |  |
| Cell 10 | 0.283 |  |  | 0.241 |  |  | |  |  |
| Cell 11 | 0.252 |  |  | 0.285 |  |  | |  |  |
| Cell 12 | 0.322 |  |  | 0.418 |  |  | |  |  |
| Cell 13 | 0.257 |  |  |  |  |  | |  |  |
| Cell 14 | 0.221 |  |  |  |  |  | |  |  |
| Cell 15 | 0.261 |  |  |  |  |  | |  |  |
| **Mean Exp 2** | **0.266** |  |  | **0.252** |  |  | |  |  |
|  |  |  |  |  |  |  | |  |  |
| **PCC values** | **Static** | | | **LSS** | | | |  |  |
| **Na_V_β3** | **LC3B** | **LAMP1** | **mTOR** | **LC3B** | **LAMP1** | **mTOR** | |  |  |
| Cell 1 | 0.329 | 0.313 | 0.339 | 0.268 | 0.655 | 0.386 | |  |  |
| Cell 2 | 0.418 | 0.608 | 0.29 | 0.423 | 0.317 | 0.282 | |  |  |
| Cell 3 | 0.371 | 0.437 | 0.309 | 0.217 | 0.333 | 0.307 | |  |  |
| Cell 4 | 0.299 | 0.483 | 0.29 | 0.414 | 0.365 | 0.176 | |  |  |
| Cell 5 | 0.17 | 0.269 | 0.309 | 0.546 | 0.518 | 0.441 | |  |  |
| Cell 6 | 0.197 | 0.455 | 0.326 | 0.435 | 0.499 | 0.385 | |  |  |
| Cell 7 | 0.237 | 0.472 | 0.417 | 0.384 | 0.505 | 0.329 | |  |  |
| Cell 8 | 0.393 | 0.419 | 0.346 | 0.296 | 0.489 | 0.359 | |  |  |
| Cell 9 | 0.332 | 0.596 | 0.319 | 0.402 |  | 0.401 | |  |  |
| Cell 10 | 0.302 | 0.309 | 0.362 |  |  |  | |  |  |
| Cell 11 | 0.354 | 0.48 | 0.43 |  |  |  | |  |  |
| **Mean Exp 3** | **0.309** | **0.440** | **0.339** | **0.376** | **0.460** | **0.341** | |  |  |
|  |  |  |  |  |  |  | |  |  |
| **PCC values** | **Static** | | | **LSS** | | | |  |  |
| **Na_V_β3** | **LC3B** | **LAMP1** | **mTOR** | **LC3B** | **LAMP1** | **mTOR** | |  |  |
| Cell 1 | 0.225 | 0.354 | 0.363 | 0.206 | 0.457 | 0.486 | |  |  |
| Cell 2 | 0.223 | 0.421 | 0.344 | 0.79* | 0.443 | 0.347 | |  |  |
| Cell 3 | 0.22 | 0.461 | 0.37 | 0.251 | 0.444 | 0.473 | |  |  |
| Cell 4 | 0.306 | 0.485 | 0.371 | 0.63 | 0.494 | 0.431 | |  |  |
| Cell 5 | 0.269 | 0.56 | 0.312 | 0.202 | 0.436 | 0.512 | |  |  |
| Cell 6 | 0.224 | 0.561 | 0.332 | 0.61 | 0.444 | 0.313 | |  |  |
| Cell 7 | 0.248 | 0.544 | 0.368 | 0.227 | 0.516 | 0.378 | |  |  |
| Cell 8 | 0.39 | 0.499 | 0.252 | 0.247 | 0.564 | 0.257 | |  |  |
| Cell 9 | 0.222 | 0.499 | 0.28 | 0.251 | 0.365 | 0.304 | |  |  |
| Cell 10 | 0.325 | 0.587 | 0.417 | 0.243 | 0.479 | 0.218 | |  |  |
| **Mean Exp 4** | **0.265** | **0.497** | **0.341** | **0.318** | **0.464** | **0.372** | |  |  |
|  |  |  |  |  |  |  | |  |  |
|  |  |  |  |  |  |  | |  |  |
| **PCC values** | **Static** | | | **LSS** | | | |  |  |
| **Na_V_β3** | **LC3B** | **LAMP1** | **mTOR** | **LC3B** | **LAMP1** | **mTOR** | |  |  |
| Mean Exp 1 |  | 0.4362 | 0.347 |  | 0.492 | 0.3589 | |  |  |
| Mean Exp 2 | 0.266 |  |  | 0.252 |  |  | |  |  |
| Mean Exp 3 | 0.309 | 0.440 | 0.339 | 0.376 | 0.460 | 0.341 | |  |  |
| Mean Exp 4 | 0.265 | 0.4971 | 0.341 | 0.318 | 0.464 | 0.372 | |  |  |
| **Mean** | **0.280** | **0.458** | **0.342** | **0.315** | **0.472** | **0.3571** | |  |  |
| **SEM** | **0.014** | **0.019** | **0.0021** | **0.035** | **0.010** | **0.001** | |  |  |
| **Supplementary Table S6. PCC values of the three independent colocalization experiments between Na_V_β3 and LC3B or LAMP1 or mTOR.** | | | | | | | |  | |
